# A reassessment of the Montmaurin-La Niche mandible (Haute Garonne, France) in the context of European Pleistocene human evolution

**DOI:** 10.1371/journal.pone.0189714

**Published:** 2018-01-16

**Authors:** Amélie Vialet, Mario Modesto-Mata, María Martinón-Torres, Marina Martínez de Pinillos, José-María Bermúdez de Castro

**Affiliations:** 1 Muséum national d’Histoire naturelle, UMR7194, UPVD, Centre Européen de Recherches Préhistoriques de Tautavel, Paris, France; 2 Centro Nacional de Investigación sobre la Evolución Humana (CENIEH), Paseo de la Sierra de Atapuerca 3, Burgos, Spain; 3 Equipo Primeros Pobladores de Extremadura (EPPEX), Casa de la Cultura Rodríguez Moñino, Cáceres, Spain; 4 Department of Anthropology, University College London, London, United Kingdom; 5 Laboratorio de Evolución Humana (LEH), Departamento de Ciencias Históricas y Geografía, Universidad de Burgos, Hospital del Rey S/N, Burgos, Spain; University of Delaware, UNITED STATES

## Abstract

We here present a comparative study of the Montmaurin-LN Middle Pleistocene mandible (Haute-Garonne, France). This mandible, of which its right and left molar series are preserved in situ, was found in La Niche cave (Montmaurin’s karst system) in 1949, and was first attributed to the ‘Mindel-Riss’ interglacial (= MIS 9 to 11) based on its geological context. Later studies based on geological and faunal evidence have attributed the Montmaurin-LN mandible to MIS 7. Following a detailed morphological and metric comparative study of the mandible in the 1970s, it was interpreted in the light of a still limited fossil record and the prevailing paradigm back then. Waiting for geochronological studies in the forthcoming years, here we review the main morphological and metrical features of this mandible and its molars, which have been reassessed in the framework of a remarkably enlarged Pleistocene fossil record since the mandible was first described, and our current, more in-depth understanding of human evolution in Europe. Using a selection of mandibular features with potential taxonomic signal we have found that the Montmaurin-LN mandible shares only a few derived traits with Neandertals. Our analyses reveal that this mandible is more closely related to the ancient specimens from the African and Eurasian Early and Middle Pleistocene, particularly due to the presence of primitive features of the *Homo* clade. In contrast, the external morphology of the molars is clearly similar to that of Neandertals. The results are assessed in the light of the present competing hypotheses used to explain the European hominin fossil record.

## Introduction

Our knowledge of settlement patterns and human evolution in Europe has increased with the emergence of new findings in recent decades [1–8]. However, there are several problems that make it difficult to put forward a coherent evolutionary scenario for the Middle Pleistocene. Numerous findings of human fossils of this period were made during the twentieth century, often by chance, with little to no stratigraphic control, and some poorly dated [9–10]. We are missing data, for example, on the precise location of important specimens like that of the Mauer mandible, the holotype of *Homo heidelbergensis*. There is also a lack of data on the geochronology of important sites such as the Montmaurin cave complex, which is the focus of the present study. Often, it is very difficult to obtain reliable and convincing geochronological data either on the particular circumstances under which the discoveries were made or the discoveries themselves (e.g. the Petralona skull), or the dating of the sediments presumably associated with the fossils (e.g. the Ceprano calvarium).

On the other hand, we could expect a largely anagenetic (linear) and coherent evolutionary sequence, so that contemporary fossils ought to present an approximately similar appearance. Thus, it has been proposed the hypothesis of a progressive 'neandertalization' of European populations during the Middle Pleistocene following a process of accretion of Neandertal features [11,12]. However, the European hominin fossil record shows a complex variability, which seems to be incompatible with a linear model. Some hominins of the mid- Middle Pleistocene share an important number of features with Neandertals, particularly in the facial skeleton and teeth, such as the Atapuerca-Sima de los Huesos (SH) hominins [13], Swanscombe [14], and Pontnewydd [15]. In contrast, other roughly contemporaneous hominins, like those of Arago, exhibit a more ‘primitive’ pattern or, in other words, a less-derived Neandertal cranial and dental morphology [16]. Similarly, the Mala Balanica (BH-1) mandible, dated to the end of the Middle Pleistocene, lacks Neandertal features [8], and the Ceprano calvarium (also dated to the mid-Middle Pleistocene), shows *H*. *erectus*-like traits [17]. The Aroeira 3 cranium, dated to MIS 11, resembles in some of its features the Arago and Ceprano specimens [18].

As a result, most scholars agree with the idea of a complex evolutionary scenario for Europe during the Middle Pleistocene, with the likely existence of refugia in the Mediterranean peninsulas [8], and the possible arrival throughout the Middle Pleistocene of different populations showing different degrees of hybridization [19]. Furthermore, the European hominin fossil record may possibly be showing different genetic processes, including drift, founder effects, and directional adaptation. Given this evolutionary context, we here present a new study of the mandible recovered from La Niche cave site of the Montmaurin karst system (Montmaurin-LN).

This specimen was first attributed to the second part of the Middle Pleistocene [20,21]. Later, and based on its biostratrigraphic and geologic contexts, the mandible was placed in MIS 7 [22], and therefore dated between 240 kya and 190 kya [23]. Vallois [20,21] was the first to briefly describe this fossil. Later, Vallois together with Billy [24,25] presented a more detailed description of the mandible. These studies, published 40 years ago, were written at a time in which our knowledge of human evolution was very different to our current one, especially given the large number of specimens that have been recovered in Europe over the past four decades since Billy and Vallois’ original publication. Although the Montmaurin-LN mandible has been studied by others [20,26,27], the information has only been used for comparative purposes as part of large database research. Therefore, besides presenting a succinct description of the main morphological and metrical features, our aim here is to assess the position of the Montmaurin-LN mandible within the African and Eurasian specimen morphospace. Knowing where the Montmaurin-LN mandible stands taxonomically and phylogenetically in relation to other specimens, and given the present paradigm, can provide further insights to the current debate on the competing evolutionary scenarios proposed to explain hominin settlement and evolution in Europe [28–33]. We are aware that the lack of radiometric dating is a limitation. The biochronological study carried out by Crégut-Bonoure et al. [22] provides a strong basis to argue for an MIS 7 origin of the human fossils from Montmaurin-LN. However, a preliminary study (in progress) of the lithic assemblage recovered from La Niche site is opening new insights on the chronology of this specimen, although always in the range of the last third of the Middle Pleistocene (MIS 9 to 6). Geochronological datings are expected to shed light on this matter. Meanwhile, we hope that the results presented here will encourage the formation of a team willing to carry out systematic excavations at La Niche cave and at other sites of the Montmaurin complex.

To sum up, assuming that the mandible recovered from La Niche cave was deposited during the last third of the Middle Pleistocene and considering the hypothesis of a linear evolution in Europe during the entire Middle Pleistocene, the Montmaurin-La Niche (LN) specimen should possess a clear Neandertal-derived morphology. In contrast, a less Neandertal-derived pattern would support the idea that a more complex evolutionary scenario took place in Europe during the Middle Pleistocene.

## The Montmaurin sites

The Montmaurin caves are located 75 km south-east of the city of Toulouse, and 19 km north of the village of Saint Gaudens (Commune de Montmaurin), Department of Haute Garonne, southwestern France (43° 13´N; 0° 36´E). The exploitation of a quarry during the twentieth century exposed a multi-level karst. Shaped by the Seygouade River, a tributary of the Garonne River, this multi-level karst comprises eight infilled cavities.

The four lowermost cavities (Le Putois) are located four metres above the level of the Seygouade River. The Coupe Gorge and La Niche cavities are located on the second level (+25 m). Finally, the Montmaurin (or Boule) and La Terrasse caves open up in the third level (+40 m). Montmaurin’s karst archaeological potential was first explored by E. Cartailhac, M. Boule, and R. de Saint-Perier at the beginning of the twentieth century [34,35]. In 1945, an archaeological survey confirmed the fossiliferous filling of the Coupe-Gorge cave, and revealed the presence of several mammal fossil remains and stone tools [36,37]. A meticulous excavation of La Terrasse cave site was led by L. Méroc between 1946 and 1961.

On 18 June 1949, Raoul Cammas recovered a human mandible among other fossil remains and some stone tools from La Niche site. Cammas also noted the presence of a human thoracic vertebra. After a short excavation done in 1980's, R. Cammas and A. Tavoso were also able to identify a human dorsal vertebra and a left tibia fragment [38].

The infilling of the La Niche cave was thought by Méroc to have come from La Terrase cave, situated just above it [39]. However, geomorphological studies of the Montmaurin karst led to the conclusion that La Niche was a cave in its own right [40]. Cammas and Tavoso [40] described three main layers: A, B, and C, from top to bottom. Layer A corresponds to the stalagmite flooring that seals the deposit. Layer B is 180 cm thick. About 100 artefacts and fossil remains were recovered from the upper part of this layer. The thickest layer C (460 cm) was further sub-divided into three sublayers: C1, C2, and C3, from top to bottom. The human remains were found in sublayer C3. According to Crégut-Bonnoure et al. [22], the absence of alluvial deposits at La Niche and the nearby Coupe-Gorge cave suggests that these cavities were filled when the Seygouade River flowed at least 15 m below these cavities, and so they estimate that the sediments and fossils may have been deposited during MIS 7, in the late Middle Pleistocene. This chronological estimation is coherent with the faunal remains recovered from La Niche cave [22], attributed to *Canis lupus*, *Ursus spelaeus-deningeri* and *Equus caballus*. The absence of cold-adapted taxa, with the exception of *Marmota*, is noteworthy. Although the implements recovered from La Niche have yet to be published, the first results emerging from D. Thiam’s on-going PhD (at UPVD, France) show it to be a lithic assemblage (n = 133) mostly comprising debitage products made on a large range of raw materials without Levallois products. The assemblage has been attributed to the late Acheulean, just as the assemblages from the other sites from the middle and upper gallery levels, Coupe-Gorge [41] and La Terrasse [42], respectively. The presence of numerous carnivore bones and tooth marks on herbivore specimens suggests that La Niche was used by carnivores as a den, and for hibernation by cave bears, alternatively with humans [22]. The pollen analyses carried out by Renault-Miskovsky and Girard [43] from four samples obtained from this site suggest a steppe environment with grasses, and a 20–35% arboreal pollen, mainly belonging to pine trees and birch.

## Materials and methods

In this study, we describe and compare the main morphological features of the Montmaurin-LN mandible and its teeth. This mandible belonged to a young adult. A CT-scan of the specimen showed that the apex of its third molars is not totally fused. Readers who wish to obtain a more detailed morphological picture of this specimen may wish to consult the publications by Billy and Vallois [24,25]. Billy and Vallois [24,25] have also, as well as other colleagues [8,44–46] already published the classic measurements for this mandible so in this study we will only present some variables when it is deemed necessary to reinforce our conclusions. Following the methodology proposed by Rosas and Bermúdez de Castro [46], we have measured the distance between the infradental (I) and the mental foramen (FOR) landmarks; the distance between FOR and the distal end of the third molar (M3); and the distance between M3 and the lingual landmark (LIN). The M3-LIN, I-FOR variables, as well as the breadth of the ramus, were corrected using the distance between the menton (MEN) and the uppermost point of the condyle landmarks (CON). In order to evaluate taxonomic and phylogenetic aspects of the Montmaurin-LN specimen, the same variables were recorded for a comparative sample of hominin mandibles (Table 1). In addition to these fossils, we have also studied a collection comprising 27 mandibles belonging to recent modern humans. These specimens are housed at the *Institut de Paléontologie Humaine*, Paris, and originate from different African countries: Aboriginals of the Canary Islands (African origin), Algeria, Dahomey, Equatorial, Democratic Republic of Congo, Guinea, Ethiopia, Gabon, Ghana Mali, Republic of Madagascar, Republic of South Africa, Mozambique, Sudan, and Tunisia.

**Table 1:** Mandibular specimens used in the present analysis.

Specimen	Location	Time Period	Reference
OH7, OH13, OH37	Olduvai (Tanzania)	Early Pleist.	[47]
UR-501	Uraha (Malawi)	Early Pleist.	[48]
KNM-ER 1802,			
KNM-ER 1484,			
KNM-ER 1501,			
KNM-ER 1502	East Turkana (Kenya)	Early Pleist.	[49]
KNM-ER 730	East Turkana (Kenya)	Early Pleist.	[50]
KNM-ER 992	East Turkana (Kenya)	Early Pleist.	[51]
KNM-ER 3734	East Turkana (Kenya)	Early Pleist.	[52]
KNM-WT 15000	West Turkana (Kenya)	Early Pleist.	[53]
KGA 10–1	Konso (Ethiopia)	Early Pleist.	[54]
KNM-BK 67	Baringo (Kenya)	Middle Pleist.	[55]
KNM-BK 8518	Baringo (Kenya)	Middle Pleist.	[56]
Thomas I	Casablanca (Morocco)	Middle Pleist.	[57]
D211	Dmanisi (Rep. Georgia)	Early Pleist.	Present study (original)
D2735	Dmanisi (Rep. Georgia)	Early Pleist.	Present study (original)
D2600	Dmanisi (Rep. Georgia)	Early Pleist.	Present study (original)
Sangiran 1b	Java (Indonesia)	Early Pleist.	[58]
Sangiran 9	Java (Indonesia)	Early Pleist.	[58,59,60]
Sangiran 22	Java (Indonesia)	Early Pleist.	[57,58]
ATD6-96	Burgos (Spain)	Early Pleist.	Present study (original)
ATD6-113	Burgos (Spain)	Early Pleist.	Present study (original)
ATE9-1	Burgos (Spain)	Early Pleist.	Present study (original)
Lantian	Chenjiavo (China)	Middle Pleist.	Present study (original)
Zhoukoudian H1	Beijing (China)	Middle Pleist.	Present study (cast) and [61]
Zhoukoudian G1	Beijing China)	Middle Pleist.	Present study (cast) and [61]
Penghu 1	Taiwan	Middle Pleist.	[62]
Hexian	Hexian (China)	Middle Pleist.	[63]
Tighenif 1	Tighenif (Algeria)	Middle Pleist.	Present study (original)
Tighenif 2	Tighenif (Algeria)	Middle Pleist.	Present study (original)
Tighenif 3	Tighenif (Algeria)	Middle Pleist.	Present study (original)
Montmaurin-LN	Montmaurin (France)	Middle Pleist.	Present study (original)
Mauer	Mauer (Germany)	Middle Pleist.	[27]
Arago II	Arago (France)	Middle Pleist.	Present study (original)
Arago XIII	Arago (France)	Middle Pleist.	Present study (original)
Atapuerca-SH			
AT-1, AT-2, AT-3,			
AT-75, AT-83, AT-172,			
AT-250+793,			
AT-300, AT, 301,			
AT-303, AT-304,			
AT-505+952+604,			
AT-511, AT-605,			
AT-888+721,			
AT-950, AT-1157,			
AT-1775, AT-1957,			
AT-2193	Burgos (Spain)	Middle Pleist.	Present study (originals) and [33,64,65]
Ehringsdorf F	Ehringsdorf (Germany)	Middle Pleist.	Maccurdy (1915), in [13]
Krapina J	Krapina (Croatia)	Middle Pleist.	[27]
Krapina H	Krapina (Croatia)	Middle Pleist.	[27]
Krapina G	Krapina (Croatia)	Middle Pleist.	[27]
La Ferrassie 1	La Ferrasie (France)	Late Pleist.	[66]
La Quina 5	La Quina (France)	Late Pleist,	Present study (original)
Regourdou	Regourdou (France)	Late Pleist.	[27]
Spy I	Spy (Belgium)	Late Pleist.	[27]
Amud 1	Amud (Israel)	Late Pleist.	[27]
Shanidar 1	Shanidar (Iraq)	Late Pleist.	[67]
Zafarraya	Zafarraya (Spain)	Late Pleist.	Present study (cast)
Bañolas	Bañolas (Spain)	Late Pleist.	[68]
Ohalo H II	Ohalo (Israel)	Late Pleist.	[69]
Pestera Cu Oase	Romania	Late Pleis.	[70]
Qafzeh 9	Qafzeh (Israel)	Late Pleist.	[27]
Skhül V	Skhül (Israel)	Late Pleist.	[27]
Cro-Magnon	Les Ezyes (Francia)	Late Pleist.	[27]
Abri Pataud	Les Ezyes (Francia)	Late Pleist.	[27]

Our study employs a phenetic approach in which specimens are grouped according to their morphological similarities. This method is related to what is known as ‘alpha taxonomy’ [71]. Mounier et al. [27] used 47 discrete morphological features in their taxonomic study of the Mauer mandible. All the features had the same weight in their analyses. The major problem with this approach is that some of the characters may lack a taxonomic signal. Some of these features may be related to sexual dimorphism or simply be reflecting the normal and idiosyncratic variability known to exist in the *Homo* genus. Last, but not least, the presence/absence of some of the features may be strongly correlated due to specific growth processes. The inclusion of these features is therefore doubling or even tripling the weight of what should in fact be considered a single trait. In short, all these characters can add noise to the analyses. Therefore, we have selected only those traits that we think, based on data from previous studies [11,12,26,60,72–77], possess a hypothetical taxonomic signal and are evolutionarily significant, and used them in this phenetic study. Nicholson and Harvati [77] have used 3D geometric morphometrics in their study of the mandibular shapes of modern humans and Neandertals. Some of their results confirm that some features are related to climatic gradients and functional specializations, thus confirming that mandibular shape is not, in itself, useful for determining population history.

On the other hand, we have not carried out a formal cladistic analysis because our study does not include a large number of taxa, and our aim is not to reconstruct the evolutionary history of the *Homo* genus. However, throughout the text (‘Results and Discussion’) we nonetheless discuss the polarity of most of the selected features using the information available to us from the fossil record. We are conscious that our phenetic analysis does not allow for phylogenetic proposals to be put forward. However, the hypothetical polarity of the chosen features enables for inferences to be made on the relationship between specimens when they share assumed derived features.

In order to explore the proximity of the specimens in terms of their morphological features, two complementary statistical analyses were performed using the R statistical software. Prior to the application of these procedures, the categorical database was transformed into a binary database. Two datasets were analyzed: the first one includes all the variables, so that only specimens preserving all morphological traits were analyzed (n = 45). In order to maximize the sample size, we analyzed a second set where the morphological features on the ascending ramus (I, J and K) were removed (S1 Table)**.** Thus, the number of specimen samples included in the analysis increased up to n = 55. The selection and removal of these features was based on a maximization-inclusion approach, as they contained the largest sample of specimens lacking these traits. The new specimens included in the latter dataset were: Arago XIII, AT-300, AT-5051, AT-888, Bañolas, D2600, D2735, La Ferrassie 1, Spy 1 and Tighenif 2.

First, we carried out a correspondence analysis using the ‘ca’ R package. The relationship between the specimens and the morphological traits was also represented. Hierarchical clustering analysis was applied subsequently by running the *hclust* function in the ‘stats’ package. We used the Ward method and binary distance. In order to evaluate the probability of every cluster of the dendrogram being supported by our data, a bootstrap analysis with 1000 replications was superimposed by running the ‘pvclust‘ package. Only approximately unbiased probabilities over 70% were highlighted.

The mesiodistal (MD) and buccolingual (BL) measurements of the Montmaurin-LN molars were taken to the nearest 0.1 mm following the techniques outlined in Flechier and Verdéne [78]. For this purpose, a special caliper with wide, flat and thin tips was used. A description of the external morphology of the molars was made following classic terminology [47,79] and using the scoring procedures of the Arizona State University Dental Anthropological System (ASUDAS) [80,81]. Dental wear was scored according to the Molnar classification [82]. Previous observations of the external morphology of the European Middle and Late Pleistocene hominins, as well as that of European fossil and recent *H*. *sapiens* samples [8,31], were used in the comparative study.

## Results

### Preservation

**Fig 1:**
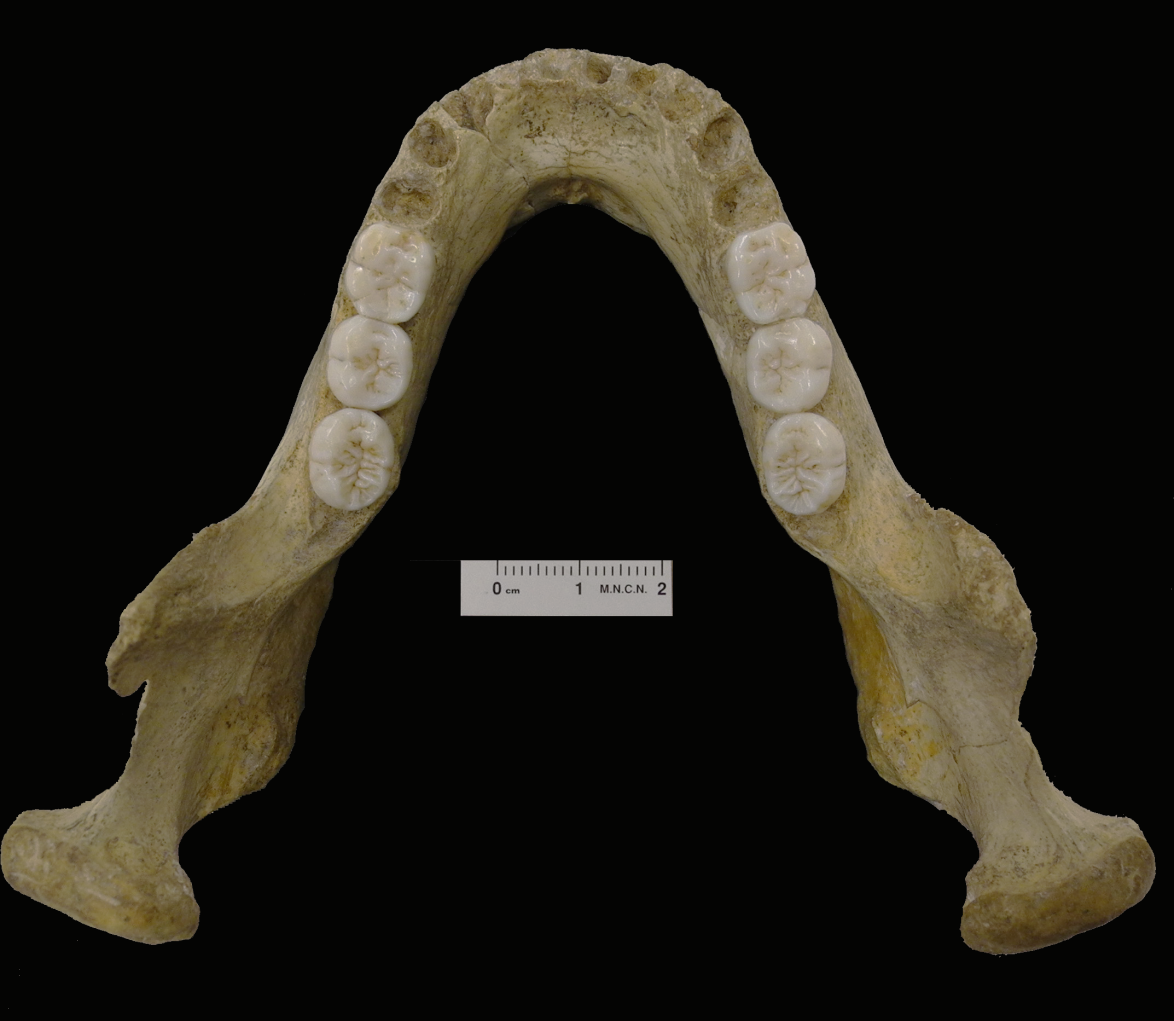
Upper view of the Montmaurin-LN mandible.

The preservation status of the Montmaurin-LN mandible is excellent. It only lacks the upper part of the alveolar wall at the level of the incisors and canines, and a small region of the internal right wall of the corpus. The anterior border of the right ascending ramus, the left coronoid process, as well as both the right and left sigmoid notches show some damage. However, the evaluation of the morphology of these parts of the mandible is not affected by this damage. Several longitudinal cracks are present in both the internal and external sides of the corpus and ramus. This cracking pattern suggests that the mandible experienced an indeterminable amount of surface weathering prior to being covered by sediments and its final deposition inside the cave. Both the right and left molar series are in situ (Fig 1) and excellently preserved.

### Comparative description

#### External aspect of the symphyseal region

The Montmaurin-LN mandible lacks any features related to the presence of a true bony chin. In fact, the symphysis profile is clearly receding, and there are no signs of a mental trigone (tuber symphyseos and lateral tubercles), mental fossae, central keel or a thickening of the inferior margin forming the so-called inverted ‘T’. Moreover, there is no incurvatio mandibulae. Billy and Vallois [24] measured the angle between a line included in the plane of the external wall of the symphysis and a basal line contained in the basal part of the corpus. They obtained a value of 105° well above a vertical profile of the symphysis. The absence of a bony chin is expected for a Middle Pleistocene fossil like this one, although some authors have observed that the mental trigone appeared early in the evolution of the *Homo* genus [47,83]. Tobias [47] concluded that the Early Pleistocene OH 7 and OH 3 mandibles exhibit both the mentum osseum and the mental trigone. Schrenk et al. [48] described the UR 501 specimen as having a distinct mental trigone and mental tubercles. Day and Leakey [50] observed a distinct mental trigone in KNM-ER 730. Kaifu et al. [59] also described a mental trigone on Sangiran 9 and Sangiran 22. In terms of the Middle Pleistocene hominins, mental trigones are present in the Tighenif 1 and Tighenif 2, as well as in the Zhoukoudian mandibles [84]. Moreover, we have observed a small mental protuberance in the Early Pleistocene mandibles from Dmanisi and in the ATE9-1 specimen from the Sima del Elefante site (Sierra de Atapuerca). However, a true chin is only present in archaic *H*. *sapiens*, like Pestera cu Oase and Zhiren 1 [70,85] and modern *H*. *sapiens* [86]. In summary, the external part of the symphysis of the Montmaurin-LN mandible is primitive, lacking any features defining the derived bony chin characteristic of *H*. *sapiens*. It is noteworthy that the Montmaurin-LN mandible shares the receding configuration of the external wall of the symphysis and a completely flat topography with most European Middle Pleistocene mandibles, except with two of the specimens from the site of Atapuerca-SH [64].

The incisura submentalis, the semilunar space beneath the inferior rim of the symphysis, is present in the Montmaurin-LN mandible. It is difficult to evaluate this feature from a cladistic point of view. It is generally present in Early and Middle Pleistocene *Homo* specimens [27]. Likewise, a more or less marked incisura submentalis is present in 16 of the 27 recent human mandibles examined in this study. In the Montmaurin-LN mandible the fossae disgastrica are facing downwards and backwards. According to Mounier et al. [27], this is a Neandertal-like pattern, which is also present in some of the Atapuerca-SH specimens (AT-605, AT-607, AT-888). It seems that the primitive condition in the *Homo* clade is to have the fossae digastrica oriented downwards, like in OH13, Dmanisi, KNM-ER 992, Sangiran or ATD6-96. A first derived condition could be a downward-backward orientation of the fossae, whereas in modern humans these fossae are generally oriented backwards. Nevertheless, the thickness of the basal surface at the level of the symphysis can be linked to the orientation of the fossae digastrica, and their possible taxonomic signal is problematic. A medial crest between the two fossae is present in the Montmaurin-LN specimen.

#### Internal aspect of the symphyseal region

The Montmaurin-LN mandible exhibits a conspicuous planum alveolare on the posterior part of the symphyseal region, measuring 11.5 mm from the alveolar border to the torus transversus superior. According to Billy and Vallois [24], the angle formed between this plane and the dental alveolar plane (i.e. the plane passing through the infradentale and the highest point on the alveolar margin between M2 and M3 [47]) is 33° wide. This angle is clearly smaller than that recorded on the Mauer (50°) or Tighenif 1, 2, and 3 mandibles (43°, 36°, and 48°, respectively), and greater than in OH13 (22°) and OH37 (25°). The presence of a marked planum alveolare is the primitive condition in the *Homo* clade, whereas an almost vertical or vertical internal wall of the posterior part of the symphysis is derived. This is the case in most Neandertals and modern humans. Interestingly, the Early Pleistocene ATE9-1 mandibles from Sima del Elefante and ATD6-96 from Gran Dolina-TD6 exhibit an almost vertical planum alveolare, sharing this feature with the G1 mandible from Zhoukoudian.

**Fig 2:**
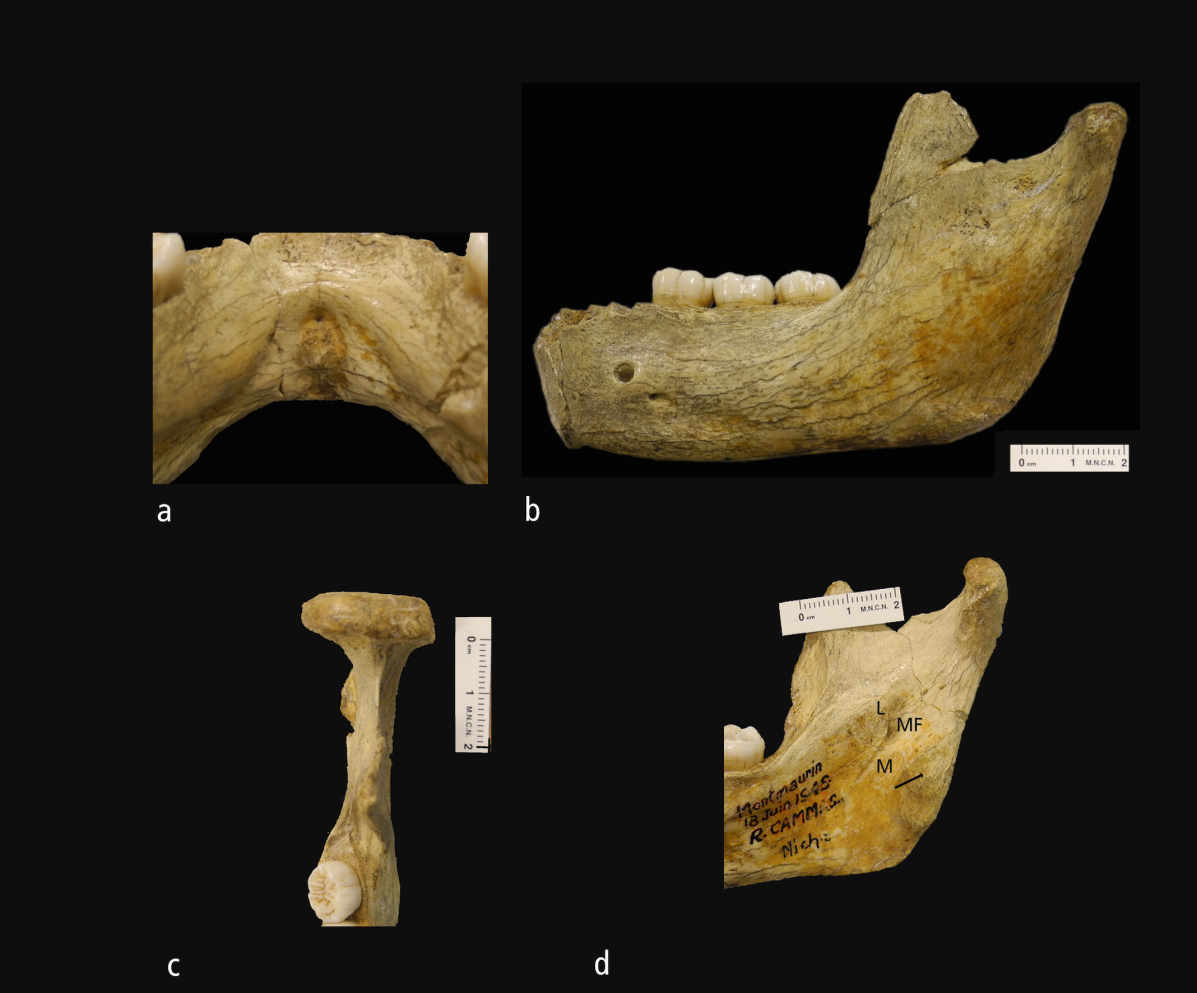
Different morphological aspects of the Montmaurin-LN mandible. a) Internal aspect of the symphysis, showing the fossa genioglossa; b) Left view of the mandible. Note the receding wall of the symphysis, the position of the mental foramen, the flat surface of the corpus and ramus, and the regular gonion profile; c) Detail of the mandible showing the medial position of the mandibular notch rim on the condylar process; d) Internal aspect of the right ramus. Note the low position of the mylohyoid line with regard to M3, the small but almost horizontal geometry of the retromolar area, the presence of a medial pterygoid tubercle (arrow), mandibular foramen (MF), the lingula (L), or the large distance between the end of the mylohyoid groove (MG) and M3. Scale bar in b, c, and d, is 2 cm.

The foramen supraspinosum is located just below the torus transversus superior, at the midline of the internal region of the symphysis and at a similar distance from the alveolar and basal borders. Billy and Vallois [10] describe it as measuring 0.7 mm and oriented downwards and backwards. According to Weidenreich [61], this foramen would be homologous to the foramina of the fossa genioglossi in great apes, and provides the only landmark for distinguishing between the alveolar and basal parts of the mandible. The fossa genioglossa is triangular in shape, with a maximum width of 7.0 mm and a height of 10.0 mm. The relief of the fossa genioglossa is well marked and with a complicated topography (Fig 2A, and see also a more detailed description in Billy and Vallois, [24]). The main relief is formed by the coalescence of the genohyoid apophyses, forming a conspicuous mental spina. The base of this fossa is limited by the torus tranversus inferior.

#### Lateral face of the corpus

The Montmaurin-LN mandible presents parallel alveolar and basal borders (Fig 2B). This configuration, as well as the slight posterior narrowing (D2735, Mauer, and AT-300), is a common feature in most Early and Middle Pleistocene mandibles. A remarkable narrowing of the corpus is only observed in D2600, whereas a variable configuration is noted in recent modern humans.

The Montmaurin-LN mandible exhibits two foramina in both the right and left sides of the corpus. The main right foramen is found below the P4-M1 position. It measures 5.6 x 3.6 mm and opens anteriorly. It is placed about 13.6 mm from the alveolar margin and 12.4 mm from the basal border. Another foramen is present on the right side. It is smaller and narrower, and placed below the M1 position. It opens posteriorly and it is placed below the main foramen. The two foramina seem to be connected and only separated by a bony bridge, which presents a longitudinal groove. On the left part of the corpus, the morphology is similar. The main foramen is also broad (4.6 x 3.4), but it is found below P4 and opens anterosuperiorly. It is placed at about 12.5 mm from the alveolar border and 15.6 mm from the basal border and opens anteriorly. The second foramen is found below M1. It is smaller and placed in a lower position and opens posteriorly. A larger bony bridge separates the two foramina. The presence of multiple foramina is not uncommon in Early and Middle Pleistocene *Homo*. Although this feature has been observed frequently in Pleistocene Asian mandibles, it is also present in African (KGA-10-1, Tighenif 1, or Tighenif 3) and European (ATD6-96, Mauer, or AT-950) specimens, as well as in Neandertals. Thus, its taxonomic signal is questionable. In contrast, an advanced position of the main mental foramen (P3, P3-P4, P4) seems to be the primitive condition for the *Homo* clade, whereas the place of this foramen under P4-M1 and M1 occurs in Neandertals and most European Middle Pleistocene hominins, including Mauer, Arago XIII, and most of the Atapuerca-SH specimens. In Early Pleistocene *Homo*, the presence of the mental foramen placed under P4-M1 is rare (KGA-10-1, SK-45).

The Montmaurin-LN mandible exhibits barely perceptible marginal anterior tubercles in both the right and left sides of the corpus. They are placed at the level of P4, like in most European Middle Pleistocene specimens. When present, the primitive condition is expressed by the presence of this tubercle at the level of C-P3 (e.g. Dmanisi D211, 2600, 2735, ATE9-1, OH-37) and P3 (KNM-ER 730, Sangiran 9), and P3-P4 (Sangiran 22, Zhoukoudian H1.

The sulcus intertoralis is weakly expressed in the Montmaurin-LN mandible and only defined by the relief of the torus marginalis superior. The relief of the prominentia lateralis is also weak and placed at the level of M2-M3/mesial M3 in both the right and left sides, respectively. The lateral prominence marks the junction of the corpus and ramus, and it is placed at the level of M1, M1-M2, or M2 in early *Homo*. Given the available fossil evidence at present, this seems to be the primitive condition. A more posterior position (M2-M3) is noted in some Early Pleistocene hominins, like KNM-ER 730, KGA-10-1, and ATD6-96, as well as in Middle Pleistocene specimens (Arago XIII, AT-172, AT-505, AT-2193, Hexian PA831, Zhoukoudian G1, H1, and K1), whereas the most pronounced expression of the derived condition is observed in Neandertals and most Atapuerca-SH specimens, where the lateral prominence is placed at the M3 level.

The Montmaurin-LN mandible does not present a true retromolar space, like Neandertals do. However, the surface behind the M3 is horizontal. It measures about 5.7 mm in both the right and left sides, far from the values published by Rosas [65] for the Atapuerca-SH mandibular sample (n = 8; X = 10.1, S.D. 2.8; 6.1–15.5). In the *Homo* genus a short and inclined retromolar area seems to be the primitive condition, whereas the most derived state is present in Neandertals [87–90]. In these hominins, there is a horizontal and large retromolar space. The presence of this feature is perhaps related (among other hypotheses) to a posterior retreat of the zygomatic and anterior ramal regions relative to a fixed molar position [91] and a shortening of the dental arcade due to a mesiodistal molar diminution [87]. In the Montmaurin-LN the M3 is not covered by the ramus, although the anterior border of the ramus is tangential to the posterior border of this molar. Thus, it is difficult to score Montmaurin-LN in a qualitative classification and to decide whether the M3 is to be recorded as uncovered or partially covered by the ramus. Following Rosas [26] and Mounier et al. [27] we have recorded the Montmaurin-LN mandible as having the M3 partially covered by the ramus. The extramolar sulcus is narrow (about 5.4 mm) compared to the width of this gutter in other hominins (Table 2). This dimension does not seem to be linked to the size of the specimen, but rather to the geometry of this anatomical region, like the direction of the molar row in terms of the position of the ramus [8].

**Table 2:** Width (in mm) of the extramolar sulcus at M3 found in some hominins.

Specimen	Width of the extramolar sulcus
OH 13	5.0
D211	9.6
D2735	10.1
D2600	8.5
UR 501	11.2
KNM-ER 992	7.0
KGA-10-1	9.0
Hexian PA831	6.0
Tighenif 1	9.5
Tighenif 2	6.3
Tighenif 3	9.9
ATD6-96	6.0
Mauer	10.0
Arago II	9.1
Arago XIII	5.1
Montmaurin-LN	5.4
AT-300	7.5
AT-505+604–952	5.6
AT-888	7.3
AT-950	6.4
Zafarraya	6.3

#### Lateral face of the ramus

The Montmaurin-LN mandible shows a clearly-defined crista ectocondylea, and a deep and wide fossa subcondylea. These features are variable in the *Homo* genus and seem to lack a taxonomic signal. The depth of the fossa masseterica is a more interesting feature. In the Montmaurin-LN mandible this region of the lateral surface of the gonian angle is flat. This feature is difficult to evaluate, although a deep masseteric relief is the more frequent condition in Early and Middle Pleistocene hominins [26]. Particularly, a deep fossa is observed in Arago XIII and in all Atapuerca-SH specimens. The fossa is flat or shallow in Mauer and Arago II. A flat surface is almost the norm for Neandertals and modern humans. On the Montmaurin-LN mandible, the border of the gonian angle shows a regular profile, which has been noted to be the most frequent shape in hominins. On the other hand, a truncated border is characteristic of Neandertals, a feature also observed in the Penghu 1, Mauer, and Thigenif 3 specimens.

Although the sigmoid notch is not well preserved in the Montmaurin-LN mandible, it is easy to estimate that the deepest point of the notch is placed in a central position (Fig 2B). Although the information is limited, it seems that a posterior position of this point could be the primitive condition, as it is present in D2735, Penghu 1, Tighenif 3, Mauer, or some of the Atapuerca-SH hominins. In contrast, a medial position of the deepest point of the mandibular notch is more common in modern humans [27]. In this study, all the modern specimens examined exhibited a medial position of this point. According to Rak et al. [75], a posterior position of the deepest point of the mandibular notch is a characteristic typical of Neandertals. The same pattern is present in most SH specimens (but see [76]).

On the Montmaurin-LN mandible, the coronoid process and the condyle are placed at the same height. The height of the condyle relative to the height of the coronoid is difficult to evaluate in the *Homo* genus from a cladistic point of view due to the limitations of the fossil record, as well as the degree of subjectivity needed when evaluating the feature [76]. Nevertheless, in some cases the height of the coronoid process relative to that of the condyle is remarkable, like in D2600, Thigenif 3 or Regourdou. According to the observations made by Mounier et al. [27], all of the Neandertal specimens in their sample possess a lower condyle with regard to the coronoid process, whereas in modern humans the height of these anatomical parts tends to be similar.

A very interesting feature of the Montmaurin-LN mandible is the medial position of the junction between the mandibular notch rim and the condyle’s articular surface (Fig 2C). The primitive condition in the *Homo* clade seems to be a lateral position of the notch relative to the condylar process, as present on the Dmanisi mandibles, KNM-ER 992, or ATD6-96. This configuration is almost the norm in modern humans, whereas a migration of the notch to a medial position (or a lateral migration of the condylar process) is a derived feature that Neandertals share with most European Middle Pleistocene specimens, except Mauer, Arago II, and AT-300.

#### Medial face of the ramus

Some variable features are present in this anatomical region. The Montmaurin-LN mandible exhibits a vertical internal coronoid pillar, and the crista endocondyloidea is weak and obliquely orientated. The planum triangulare, which is placed between the internal coronoid pillar and the crista endocondyloidea, is deep and well defined (Fig 2D). This mandible shows a conspicuous lingula mandibulae and a diagonal mylohyoid groove. Rosas and Bermúdez de Castro [92] have previously drawn attention to this trait, highlighting both the angle of inclination and the position of its distal end. The lack of information in the literature prevents us from carrying out a thorough evaluation of this feature. In any case, it is interesting that the Montmaurin-LN mandible shares this same configuration with all European Middle Pleistocene specimens and Neandertals. The distal end of the mylohyoid groove is located in a more posterior position with regard to M3 than is the case in African and Asian specimens such as OH 13, KNM-ER 730, OH 22, KNM-BK 67, Tighenif 1, Tighenif 3, Sangiran 9, and Zhoukoudian H [91]. In the Montmaurin-LN mandible a bony bridge covers most of the trajectory of the mylohyoid groove (Fig 2D).

The pterygoid fossa is deep in the Montamaurin mandible. This is considered a characteristic feature of Neandertals, although it is not exclusive to this group. Thus, a deep pterygoid fossa is observed in OH13 [47], Sangiran 9 [27], Tighenif 3, and Arago II. A deep pterygoid fossa is infrequent in modern humans [27]. The Montmaurin-LN mandible exhibits a well-developed medial pterygoid tubercle, especially on the right side. This feature has been considered as being characteristic of Neandertals [93,94], whereas it is absent in *Australopithecus* and *Paranthropus* [95]. The medial pterygoid tubercle is located just above the gonial region, approximately at the level of the alveolar plane. It is triangular in shape and the base coincides with the posterior border of the ramus. It is important to avoid confusing it with the *tuberculum pterygoideum inferius* which, when present, is found just above the medial pterygoid tubercle and forms the lower limit of the *sulcus colli* [61]. This feature is not present in the Montmaurin-LN mandible. An exhaustive study of the medial pterygoid tubercle [96] confirms that this feature is very frequent in Neandertals (ca. 89%), as well as in the Atapuerca-SH mandibles (ca. 55%). However, it is not exclusive to Neandertals and Middle Pleistocene hominins, since it is also present in specimens such as SK15, KNM-BK 67, Xujiayao 14 [97], and some archaic *H*. *sapiens* such as Ohalo 2 H2, Pestera cu Oase 1, or Predmosti 3. Likewise, we have noted a well-developed medial pterygoid tubercle in 11 out of the 27 (40.7%) African modern humans examined in this study. Above the medial pterygoid tubercle, there are two barely expressed ridges for the insertion of the pterygoid muscle.

#### Medial face of the corpus

The main feature to describe in this region of the mandible is the mylohyoid line. This line is well-marked and slightly inclined on the Montmaurin-LN mandible. It is in a low position in terms of the more distal part of the M3: 9.3 mm on the right side, and 8.1 mm on the left side. These measurements are small in comparison to those from the Atapuerca-SH specimens, which are generally well above 10.0 mm [65]. However, in these specimens the M3 is displaced anteriorly and the mylohyoid line starts well behind the M3. The taxonomic signal of this feature seems to be low, according to the data compiled by Mounier et al. [27]. In contrast, the geometry of the mylohyoid line shows a well-defined polarity in the *Homo* genus. It is faint and practically absent in most early *Homo* specimens [98], like in D 211 and D 2735. A parallel, subparallel, or only slightly inclined mylohyoid line is the most common pattern observed in the *Homo* clade. Thus, the Montmaurin-LN mandible is similar to most *Homo* mandibles, including those of Arago, Mauer, Tighenif, Zhoukoudian, ATD6-96, and to most of archaic and modern humans. However, the mylohyoid line is steeply inclined (diagonal) in Neandertals and the Atapuerca-SH mandibles (except AT-950) ending around the digastric fossa area. Interestingly, this line is diagonal in the D 2600 specimen. In the Montmaurin-LN mandible the mylohyoid line separates a smooth planum subalveolaris from a deep subalveolar fossa. At the level of M1 the mylohyoyd line merges with a marked alveolar prominence (Fig 2A), which ascends until it merges with the torus transversus superior.

#### Some metric features

The total length of the Montmaurin-LN mandible, measured by Billy and Vallois [24], is 110 mm (Table 3). This value is small in comparison to other Early and Middle specimens [5]. This measurement is not far from those of the Arago 2, Regordou, Zafarraya or Zhoukoudian H1 specimens [5]. If we consider the MEN-CON length, the value (123.4 mm) obtained by Rosas and Bermúdez de Castro [46] is small and clearly below the values of large Middle Pleistocene specimens like Mauer (134.3 mm), Tighenif 3 (143.3 mm), Arago XIII (142.0 mm), AT-605 (139.3 mm), or AT-888 (138.0 mm). The height and thickness of the corpus at the M1 level are also small, and very similar to those of the ATD6-96 specimen. The robustness index is not particularly high, and similar to those of European Early and Middle Pleistocene hominins [99].

**Table 3:** Some measurements and indices of the Montmaurin-LN mandible.

Total length ^1^	110.0 mm
MEN-CON^2^	123.4 mm
Ramus breadth^2^	39.4 mm
Ramus breadth/total length (X 100)	35.8 mm
Corpus height, M1 level	28.5 mm
Corpus breadth, M1 level	16.0 mm
Robustness index	56.1
Infradental-Foramen (I-FOR)^2^	36.0 mm
Foramen-M3 (FOR-M3)^2^	42.6 mm
M3-Lingula (M3-LIN)^2^	27.8 mm
M3-LIN/Total length (X 100)	25.3
M3-M3 (buccal-buccal) distance^3^	68.9 mm
M3 (lingual)-Infradental (M3-I)^2^	66.0 mm
Index of the M3-M3/M3-I alveolar arcade	96.2

1. According to Billy and Vallois [24].

2. According to Rosas and Bermúdez de Castro [46].

3. According to Rosas and Bermúdez de Castro [98]

The ramus breadth is in the lower part of the range for other European Middle Pleistocene specimens. However, the size relationship between the ramus breadth and the total length is 35.8. This value is similar to that for Mauer and is beyond the range of variation for Neandertals and the Atapuerca-SH hominins [46]. The I-FOR distance in the Montmaurin-LN mandible is small and also beyond the European Middle Pleistocene range (including Mauer). The FOR-M3 distance in the Montmaurin-LN mandible, 42.6 mm, falls within the range for other European Middle Pleistocene hominins, but out of that for the Atapuerca-SH specimens and Neandertals. Although this distance does not show the primitive condition of the African Early and Middle Pleistocene *Homo*, it is not far from the minimum range for the East African Middle Pleistocene mandibles [46]. The M3-LIN distance in the Montmaurin-LN mandible, 27.8 mm, is not informative, since it falls within the variation range recorded for the Tighenif specimens and European Middle Pleistocene hominins. This measurement of the Montmaurin-LN mandible is beyond the range known for Neandertals [46]. When the M3-LIN distance is corrected by the total length, the value of the index (25.3) is similar to that of Mauer and at the lower range of the Atapuerca-SH and Neandertal samples.

Lastly, the length/width index of the Montmaurin-LN mandible (96.2) is high and similar to those recorded for the Mauer or Arago II specimens, but lower than those obtained for the Atapuerca-SH sample and the Neandertals. Having said this, the length/width index for Montmaurin stands below the 100.00 limit characteristic of African Early Pleistocene mandibles and the Dmanisi specimens [98].

#### Statistical analyses

S1 Fig shows the biplots for the correspondence analyses of dimensions 1 and 2. It also shows the inertia percentage for all of the dimensions in the dataset comprising all the variables or those with a reduced number. For the first dimension both datasets display inertias of 33.2% and 30.3%, respectively. The second dimension has inertias of 10.8% and 12.5%, respectively. Overall, the inertias for the first two dimensions are practically interchangeable (44.0% and 42.8%) showing that the removal of variables I, J, and K barely alters these percentages. Moreover, according to the average rule and in order to discuss the statistical results of the correspondence analysis, it would be necessary to take into account nine dimensions in both datasets (S2 Fig). An image with nine dimensions is visually unmanageable and we acknowledge the fact that the inertias of the first two dimensions are relatively low, as they do not even reach the 50% cutoff. So, the interpretation of these results should be considered with some caution as more than half of the variation remains unexplained. For this reason, a hierarchical clustering analysis was applied subsequently in order to complement and improve the interpretations.

Dimension 1 generally separates early and recent modern humans on the right (positive values) whereas Neandertals and Early and Middle Pleistocene hominins occupy the left half (negative values). The closer the variables and the specimens are in the biplot, the closer the relationship between them. For instance, modern humans tend to be found closely packed in a limited space of the biplot, being placed in the positive area of dimension 1, and either in the positive or the negative area in terms of the second dimension. The features appearing in the same area as modern humans include, among others, D1, E2, G2, J1, K2, L1. By comparing these particular traits with the real ones in S1 Table we can note that most modern humans present these features.

The Montmaurin-LN mandible is close to those of Middle Pleistocene hominins. Clearly, dimension 2 behaves well when Neandertals can be separated (either the positive or negative area depending on the biplot of S1 Fig) from other Early and Middle Pleistocene hominins (situated in the opposite area). As mentioned earlier, these interpretations must be complemented by another statistical approach in order for the complete variation to be included and fully appreciated.

**Fig 3:**
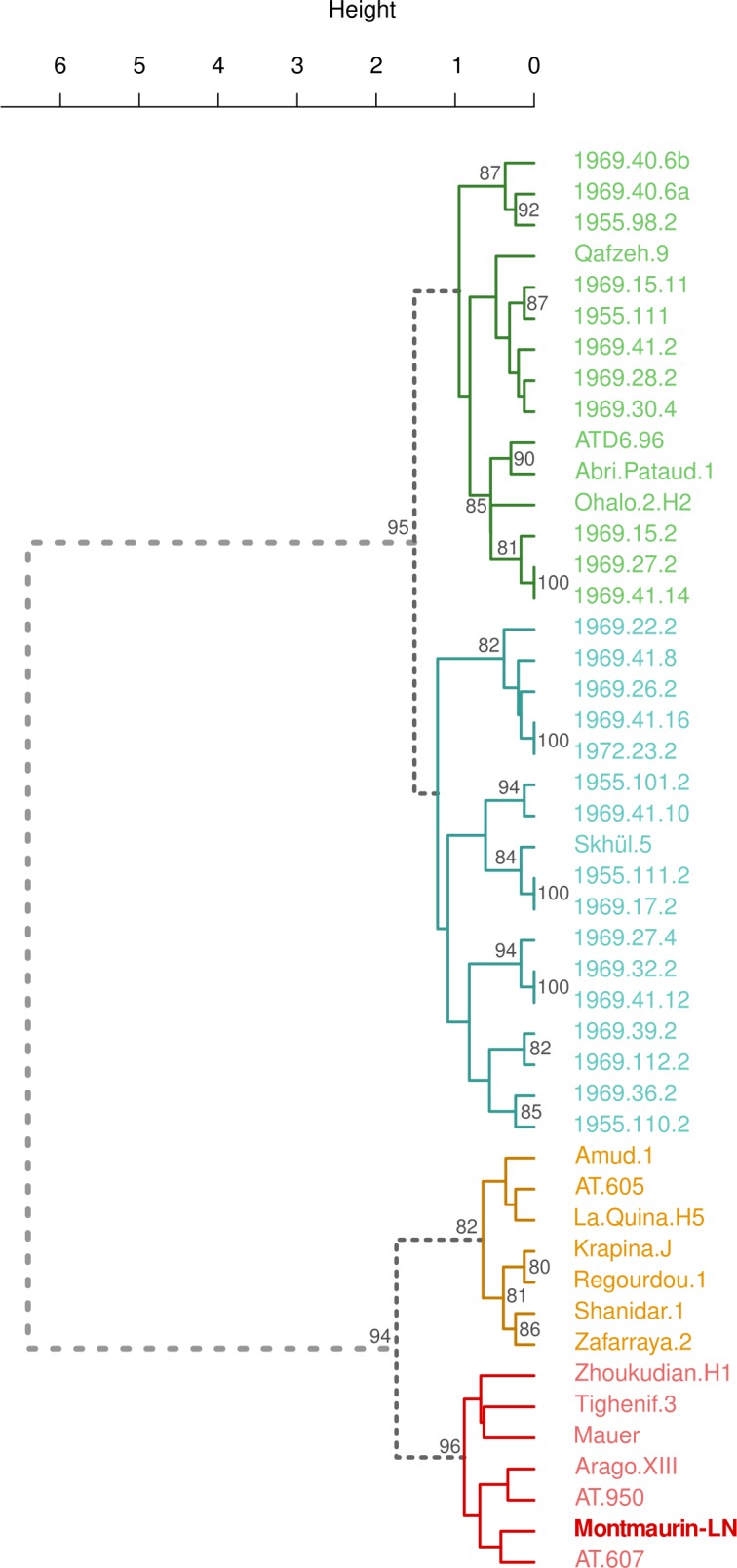
Dendrogram showing the results of the clustering analysis, when we include all morphological traits used in this study. Ward method and binary distance are employed. Only bootstrap probabilities (in blue) over or equal to 70% are shown.

**Fig 3** shows the cluster from the dataset that contains all variables, whereas S3 Fig depicts the cluster from the dataset with the fewer variables. The two dendrograms clearly differentiate two main groups, which are firmly supported by bootstrap probabilities (≥ 85%): 1) recent and early modern humans, plus ATD6-96; and 2) Neandertals and Early and Middle Pleistocene hominins (including Montmaurin-LN).

The group in which the Montmaurin-LN mandible is included presents two sub-subclusters: 1) the Neandertal specimens (La Quina H5, Regourdou, Krapina, Shanidar, Zafarraya, La Ferrassie 1, Spy 1 and Bañolas); and 2) the Early and Middle Pleistocene hominins. This latter group is where the Montmaurin-LN mandible is found. The Atapuerca-SH specimens can be found in both clusters. In terms of bootstrap probabilities, in the dendrogram with all the variables, both subclusters present relatively high bootstrap probabilities: 91% for Neandertals, and 91% for the Early and Middle Pleistocene hominins. These values are slightly smaller in the dataset comprising less variables (81% and 73%, respectively), which would imply that the variables that were removed are useful for establishing differences between Neandertals and more ancient populations and/or species. Taking into account that Montmaurin-LN clusters within the ‘non-Neandertal’ group with a probability of 91% it is clear that this mandible is more closely related to the Early and Middle Pleistocene populations than to Neandertals.

Recent modern humans, archaic *H*. *sapiens* (Qafzeh 9, Abri-Pataud 1, Ohalo 2, Skhül 5), and, surprisingly, ATD6-96 are all found in the same subcluster. Two further sub-subclusters are identified in this group, presenting bootstrap probabilities below 70%.

#### Dimensions

The MD and BL dimensions of the M1 and M2 of the Montmaurin-LN mandible are similar to those of the Atapuerca-SH and Neandertal (excluding Krapina) samples, as well as those of the Mala Balanica specimen (Table 4). The M1 and M2 from Krapina are particularly large, and are considered as a separate sample. The most relevant of the Montmaurin-LN molar dimensions is the clear M1>M2 size sequence. The Atapuerca-SH and Neandertals (except Krapina) samples, as well as the Mala Balanica specimen exhibit an M1 = M2 size sequence. Meanwhile, other European Middle Pleistocene hominins, as well as the Krapina sample, possess an M1<M2 size sequence. The size relationship between M3, and M1 and M2 is largely variable, including remarkable reductions in some specimens from the Atapuerca-SH sample and Arago 2, and a relatively larger size like in the case of the Montmaurin-LN specimen.

**Table 4:** Measurements (in mm) of the mesiodistal (MD) and buccolingual (BL) dimensions of the permanent lower molars of the Montmaurin-LN, Mala Balanica (BH-1), Arago 2, Arago 1, and Mauer specimens, and descriptive statistics for these teeth from the Atapuerca-SH, Neandertals (except Krapina), and Krapina hominin samples.

Montmaurin-LN			M1	M1	M2	M2	M3	M3		
			MD	BL	MD	BL	MD	BL		
	Right		11.7	10.6	11.0	10.9	12.8	10.3		
	Left		11.8	10.7	11.0	11.0	12.7	10.5		
		**M1**			**M2**			**M3**		
		**N**	**Mean**	**SD**	**N**	**Mean**	**SD**	**N**	**Mean**	**SD**
Atapuerca-SH^1^	MD	39	11.2	0.5	39	11.1	0.5	38	11.3	0.8
	BL	38	10.4	0.4	39	10.2	0.7	38	9.8	0.7
Neandertals^1^	MD	33	11.5	0.7	26	11.6	0.7	25	11.4	0.9
(except Krapina)	BL	34	10.8	0.6	26	10.9	0.7	26	10.9	1.1
Krapina [100]	MD	14	12.4	0.8	12	12.7	0.8	11	12.1	0.7
	BL	14	11.5	0.8	12	11.5	0.7	10	10.8	0.4
BH-1^2^	MD		11.5			11.5			12.1	
	BL		10.9			10.9			10.5	
Arago 2^1^	MD		11.0			11.9			10.5	
	BL		10.9			10.9			9.7	
Arago 13^1^	MD		13.8			14.6			13.2	
	BL		13.0			13.9			12.5	
Mauer^3^	MD		11.6			12.7			12.2	
	BL		11.2			12.0			10.9	

1. Measurements taken by JMBC

2. From Roksandic et al. [8]

3. From Howell [84]

#### Morphology

First molars. The occlusal contour of the Montmaurin-LN first molars (M1s) has the shape of a rounded rectangle that is mesiodistally elongated (Fig 1)**.** On the right M1 small dentine patches are observed on the protoconid, metaconid, and entoconid cusps, whereas on the left M1 small patches are observed on the protoconid, entoconid and hypoconulid cusps (grade 3 of the Molnar classification). These teeth exhibit five well-developed cusps. The trigonid (protoconid and metaconid) is larger than the talonid (hypoconid, hypoconulid, and entoconid). Both the right and left M1s exhibit a well-developed middle trigonid crest (ASUDAS grade 2). That is, there is a remarkable crest connecting the mesial aspects of the protoconid and metaconid. The anterior fovea is pronounced and linear on both the right and left M1s (grade 2). On the left M1 the fovea is open toward the mesial marginal ridge by means of a sinuous central groove. A well-developed anterior fovea is characteristic of the Atapuerca-SH hominins and the Neandertals [31,101], with percentages in excess of 80%. Other Middle Pleistocene hominins (Arago, Mauer, and Pontnewydd) exhibit a grade 2 anterior fovea (43%), which is absent in the Mauer and Mala Balanica (BH-1) specimens. A well-developed anterior fovea is very rarely seen on the fossil and recent European *H*. *sapiens* samples [31]. Concerning the middle trigonid crest, it is present practically on all of the Atapuerca-SH specimens, on 57% of other Middle Pleistocene specimens, and on 90% of the Neandertals [31]. The combined expression of anterior fovea and middle trigonid crest has been considered as being typical of Neandertals [101,102] and European Middle Pleistocene populations [31]. The mesial marginal ridge is broad on both Montmaurin-LN M1s exhibits some small enamel crenulations on the left antimere. Both the right and left M1s also show a distal trigonid crest (grade 1) in the shape of a prolongation of the accessory distal ridge of the protoconid and metaconid cusps. This feature is less common on the European Middle Pleistocene hominins. Six out of a total of 20 Atapuerca-SH M1s present a distal trigonid crest (mostly grade 2), whereas this feature (grade 1) is only present on two of the other six Middle Pleistocene hominins. The different degrees of expression of the distal trigonid crest are present on 52% of the Neandertals and about one third of the European fossil and modern human *H*. *sapiens* samples [31]. No deflecting wrinkle is present on the M1s of the Montmaurin-LN specimen, and the conspicuous essential crest of the hypoconid establishes a clear contact with the metaconid (Y-pattern). Interestingly, the Y-pattern is present on about 56% of all Middle Pleistocene hominins (Atapuerca-SH, Arago, Pontnewydd, Mauer, and Mala Balanica), whereas it is very frequent (82%) in Neandertals and in the fossil and recent European *H*. *sapiens* samples studied by [31]. The hypoconulid is large or very large in relation to the other cusps of the talonid (grade 5); it is distobuccally placed, and well delimited by deep fissures. No traces of the C6 (entoconulid), C7 (metaconulid or tuberculum intermedium), nor protostylid are visible on these molars. The C6 is a constant feature on the Atapuerca-SH hominins, and very frequent on other Middle Pleistocene hominins, Neandertals and *H*. *sapiens* [31], whereas the C7 is present on BH-1 [8]. The protostylid may be useful when distinguishing between populations, rather than being a taxonomic marker in itself [103]. The distal fovea is also absent on the Montmaurin-LN M1s.

Second molars. The occlusal contour of the Montmaurin-LN M2s is a rounded square, with similar MD and BL dimensions (Table 4). The right M2 shows a bucco-lingually broader trigonid relative to the talonid (Fig 1). Only the enamel is slightly worn (grade 1 of the Molnar classification). On both the right and left M2s four cusps are observed. A small distal groove, placed at the disto-buccal corner on the right M2 suggests that the hypoconulid was fused with the hypoconid during development, but the fifth cusp is not expressed (ASUDAS grade 0). 33%, and 14% of the Atapuerca-SH and Neandertals M2s, respectively, either lack a hypoconulid cusp or have it minimally expressed. The hypoconulid is always present on other Middle Pleistocene M2s (Arago, Mauer, Mala Balanica, and Pontnewydd), whereas its absence is common on fossil and recent *H*. *sapiens* [31]. The mesiodistal size of the trigonid of the Montmaurin-LN M2s is roughly similar to that of the talonid. Both the right and left M2s show a well-developed linear anterior fovea and a well-developed middle trigonid crest. On the right M2 the fovea is open toward the mesial marginal ridge by a central groove. As in the case of the M1s, the anterior fovea is very frequently found on the Atapuerca-SH hominins (76%), other Middle Pleistocene hominins (87%), and Neandertals (95%), whereas its presence on *H*. *sapiens* is less than 50% [31]. The expression of an uninterrupted middle trigonid crest is very frequent on the Atapuerca-SH hominins (90%) and Neandertals (74%), but it is only present in this shape on 28% of other Middle Pleistocene hominins (Arago 6 and Arago 10). The combined expression of grade 2 anterior fovea and a middle trigonid crest is frequent on the Atapuerca-SH hominins (71%) and Neandertals (67%). It is less common on other Middle Pleistocene hominins (25%) and practically absent on *H*. *sapiens* [31]. This trait combination has been described as typical of *H*. *neanderthalensis* and European Middle Pleistocene hominins [31,101]. As in the case of M1, this combination is absent on the Mauer and BH-1 M2s. The distal trigonid crest is absent on the left Montmaurin-LN M2, but a minimum expression of this feature is observed on the right antimere (ASUDAS grade 1). This crest is interrupted by the central groove. The distal trigonid crest (grade 2) is rarely expressed on Middle Pleistocene hominins (one out of nine), Neandertals, and *H*. *sapiens*, but grade 1 is observed on 50% of Neandertals, and on about 33% of other European hominins [8, 31]. A deflecting wrinkle is missing on the M2s, and the well-developed essential crest of the hypoconid is in contact with the metaconid (Y-pattern). This pattern is present on about 45% of the Middle Pleistocene hominins and the European fossil *H*. *sapiens*, whereas it is present on 71% of the Neandertals [31]. The C6, C7, protostylid, and distal fovea are absent on the M2s.

Third molars. The occlusal contour of the Montmaurin-LN M3 molars is a mesiodistally elongated rounded rectangle (Fig 1). Only the enamel of the trigonid is slightly affected by wear (grade 1 of the Molnar classification). The morphology of these teeth is complicated by the presence of accessory ridges, particularly on the entoconid. The hypoconulid cannot be easily distinguished from the hypoconid, but an enamel fold placed at the distolingual corner suggests the presence of a fifth cusp, particularly on the right M3 (ASUDAS grade 2). The total absence of the hypoconulid (grade 0) is not infrequent on the Atapuerca-SH hominins (6 out of 23 = 26%), Neandertals (2 out 16 = 12.5%), and on the European fossil (33%) and modern (50%) samples [31]. This cusp is always present on other Middle Pleistocene hominins. On the Montmaurin-LN specimen, an enamel wrinkle of the entoconid area suggests the presence of a small C6 (ASUDAS grade 1). The trigonid area is clearly greater than the talonid area. Again, a remarkable middle trigonid crest and a linear anterior fovea are present on these teeth. On the left M3 the fovea is open towards the broad mesial marginal ridge by a central groove. As in the M1s and M2s the combined presence of anterior fovea and middle trigonid crest is characteristic of the Atapuerca-SH and *H*. *neanderthalensis* (57% and 65%, respectively) specimens. This pattern is not present on other Middle Pleistocene specimens (Arago, Mauer, and Mala Balanica), and it is absent on the fossil and recent European *H*. *sapiens* samples studied by Martinón-Torres et al. [31]. On both Montmaurin-LN M3s a distal trigonid crest (ASUDAS grade 1) is present. It is interrupted by the central groove. This feature is prevalent on the Atapuerca-SH hominins (69%) and other Middle Pleistocene specimens, but it is less frequent (38%) on Neandertals. A deflecting wrinkle is missing from the Montmaurin-LN M3s. On the right M3 the four main cusps are in contact (plus-pattern), whereas on the left M3 there is minimal contact between the accessory ridge of the protoconid and the accessory ridge of the entoconid (X-pattern). This fissure pattern is predominant on the Middle Pleistocene M3s, but the Y-pattern is present on 60% of the Neandertal M3s [31]. No C7, protostylid, and distal fovea are present on the Montmaurin-LN M3s.

## Discussion

The Montmaurin-LN mandible could have belonged to a female. Billy and Vallois [24] also considered this possibility, although they acknowledged the fact (p. 279) that not enough comparative information was available to support this conclusion. A single specimen does not allow knowing the possible sexual dimorphism of the population to which it belongs. However, thanks to the increase in the number of specimens in the Middle Pleistocene fossil record, and especially the mandibular sample obtained at the site of Atapuerca-SH, it is clear that the size of the Montmaurin-LN mandible falls on the lowest part of the variation range. Therefore, the possibility that this mandible belonged to a female is worthy of consideration. This could explain the absence or weak expression of some specific morphological features, such as the sulcus intertoralis, the prominentia lateralis, or the crista ectocondyloidea. None of these features has a taxonomic signal and does not affect our evaluation of the Montmaurin-LN mandible. It is interesting to mention that in the large mandibular sample from Atapuerca-SH there is a considerable similarity in the expression of the diagnostic features included in this study [26]. That is, if both males and females are represented in the Atapuerca-SH mandibular sample [104] we can confidently assume that sex does not influence in the appearance of the features considered as Neandertal apomorphies.

In the present study we have noted that the Montmaurin-LN mandible shares some features with Neandertals and European Middle Pleistocene specimens. The lateral prominence is located on the M3 position, a derived feature for the *Homo* clade. It is important to note that this feature is very variable in modern humans. Thus, 6 out of 27 modern specimens of the sample included in this study also show a lateral prominence placed at the M3 level. The medial junction of the mandibular notch and the condylar process of the Montmaurin-LN mandible are also noteworthy. This is a derived feature shared with Neandertals and most of the Atapuerca-SH specimens. This same position is only noted on Tighenif 1, whereas it is in a lateral position on Mauer, Arago II, AT-300, and Spy 1. As stated above, the small retromolar area of the Montmaurin-LN mandible is horizontal. However, a horizontal retromolar area is also observed on Tighenif 1 and Skhül 5. Finally, it is interesting that the distal end of the mylohyoid groove of the Montmaurin-LN mandible is located in a posterior position, like in all European Middle Pleistocene specimens and Neandertals, whereas a more forward position is noted on the African and Asian specimens [92]. The presence of the medial pterygoid tubercle on the Montmaurin-LN mandible ought to be considered with some caution. This feature has been regarded as typically Neandertal [94]. This is certainly a correct assumption, although its presence on some African and Eurasian Early Pleistocene specimens as well as on modern humans makes it difficult to define the polarity of this feature. Therefore, the presence/absence of the medial pterygoid tubercle should be treated with caution.

In terms of the teeth, the external morphology of these elements on the Montmaurin-LN clearly places this specimen alongside the Atapuerca-SH hominins and the Neandertals. In particular, the concomitant expression of a continuous middle trigonid crest and a large anterior fovea on the three molars is noteworthy. Furthermore, the absence of hypoconulid on the M2s and the weak expression of this cusp on the M3s is also remarkable. The derived Neandertal condition of the Montmaurin-LN molars is undeniable.

In contrast, the Montmaurin-LN mandible exhibits a large list of plesiomorphies for the *Homo* clade, including the total absence of features related to the bony chin, an anterior position of the main mental foramen, a partially covered M3, a small retromolar area, a regular gonion profile, a shallow pterygoid fossa, a subparallel mylohyoid line, or a marked planum alveolare. The I-FOR distance is small in the Montmaurin-LN mandible. According to Rosas and Bermúdez de Castro [46], a large I-FOR distance in European Middle Pleistocene hominins may be as a result of the backward position of the mental foramen and possibly of the enlargement of the anterior part of the mandible, linked to the expansion of the anterior teeth. If this is the case, the Montmaurin-LN mandible preserves the primitive condition observed in earlier *Homo* specimens [46]. Interestingly, other measurements and indices obtained for the Montmaurin-LN mandible are out of the known ranges for Neandertals and the SH sample.

The correspondence and clustering analyses show that Neandertals and modern humans are grouped in well-separated clusters. The Montmaurin-LN mandible falls under the same group as the Middle Pleistocene hominins, alongside some of the Atapuerca-SH specimens. The mandibles of the Atapuerca site cluster either with Neandertals or with other Middle Pleistocene mandibles, depending on their greater or lesser number of derived Neandertal features.

To summarise, the Montmaurin-LN mandible shares only two (or three, if we take into account the medial pterygoid tubercle) morphological features with the Neandertals, together with a definitive Neandertal dental pattern. Given the assumed chronology of this specimen, a more Neandertal configuration was expected for the Montmaurin mandible if we assume an evolutionary continuity of the Middle Pleistocene populations from at least 600 kya (the presumed chronology of the Mauer mandible [105]) until the appearance of the Neandertals at the end of the Middle Pleistocene and the Late Pleistocene [28]. Against this background, *H*. *heidelbergensis* and *H*. *neanderthalensis* would represent two chronospecies forming a single reproductive continuum in Europe [28]. This same idea is also considered in the so-called two-phase model for the origins of Neandertals [106]. According to this model, a speciation event could have taken place at the end of the Middle Pleistocene. This event would have occurred due to changes in the dynamics of craniofacial growth, probably leading to a morphology of the mandible with most (if not all) of the main Neandertal features. If the Montmaurin-LN mandible belongs to the late Middle Pleistocene, our results would not support this model.

The accretion model [11,12,107] also supports the idea of evolutionary continuity in Europe. This model explains evolutionary changes in the Middle Pleistocene European populations as a consequence of the gradual accumulation of distinctive morphological features. The shift in the frequency of non-metrical features would have culminated in a lower variability in their expression. Eventually, these features would be fixed in late *H*. *neanderthalensis*. That is, the later the population, the greater the number of Neandertal characteristics present in its morphology. The process behind the accretion model would be genetic drift, resulting from a small and isolated population size. As in the two-phase model, the Montmaurin-LN jaw does not fit into the accretion model if its chronology is actually set in the late Middle Pleistocene.

The models of continuity are difficult to test due to the limited and discontinuous nature of the fossil record, as has been noted by Hublin [11]. Furthermore, the finding of two enigmatic specimens, the calvaria of Ceprano (Italy), and the BH-1 mandible from Mala Balanica (Serbia) have further complicated the Pleistocene evolutionary scenario in Europe [8,17,108]. The BH1 mandible, dated to the late Middle Pleistocene, does not present Neandertal features [8]. Although we agree that this specimen exhibits a clear primitive pattern, its fragmentary nature suggests we should be approaching it with caution. We also know that Neandertal traits are randomly expressed in some (but not all) Middle Pleistocene specimens. Concerning the Ceprano calvaria, the primitive aspect of this fossil is also at odds with its new younger datation [7]. In order to test the accretion or the two-phase models we cannot ignore these fossils. Moreover, the fact that the Montmaurin-LN mandible exhibits a more primitive aspect than the supposedly older Atapuerca-SH hominins is worthy of consideration.

Most European Middle Pleistocene hominins exhibit similarities in their cranial and dental morphology, evincing a common phylogenetic history. However, they cannot be aligned in a chronological sequence of progressive ‘neandertalization’ [10,31]. For instance, it is interesting to note the morphological disparity between the Arago and Atapuerca-SH hypodigms, despite their rough similar chronology. Atapuerca-SH hominins exhibit dentognathic and facial similarities with Neandertals [27], whereas the teeth, mandibles and cranial bones of the Arago hominins show a more primitive morphology and only a few Neandertal traits. Thus, the Arago dental features have been interpreted either as the result of the influence (hybridization) of earlier populations [109] or by the presence of a *H*. *erectus* subspecies in Europe [16]. The Montmaurin-LN mandible represents a third model, in which teeth exhibit a definitive Neandertal pattern, whereas primitive features predominate in the mandible. All this evidence supports the idea of an unclear boundary between the so-called pre-Neandertal populations and the Neandertals of the Late Pleistocene, and thus the difficulties encountered when defending the hypothesis of a linear evolution in Europe. The Mala Balanica specimen [8] is the best example of this. It is not possible to affirm that from a certain point in time the fully fixed Neandertal morphology was present in Europe. Given all these uncertainties, it seems more coherent to speak about a Neandertal clade, with some possible lineages sharing a common ancestor in the Middle Pleistocene.

In this regard, the presence of some Neandertal-like features on the Gran Dolina-TD6 Early Pleistocene hominins advocates either for a phylogenetic continuity between the Early and Middle Pleistocene populations from Europe, or for a common origin for both [19]. Thus, we propose that the colonization of Europe may have been the result of several hominin migrations originated from a central area of dispersals in Eurasia (the so-called CADE), located in the Levantine Corridor and continuously inhabited by a ‘source population’ [110]. Southwestern Asia would have represented a crossroads between Africa and Eurasia and a true hotspot for biodiversity during the entire Pleistocene [111,112]. Evolutionary changes, as well as intermittent African influences, may have occurred in the source population and were reflected in the demes that migrated to the east and west of the vast Eurasian continent [19,113]. Europe would have worked as an open system, experiencing numerous episodes of immigration from western Eurasia sources [10]. The timing and success of these dispersals were probably significantly restricted by climatic and environmental conditions [10,114–119], resulting in a non-linear and discontinuous settlement of the European continent with frequent population wipe-outs, local extinctions and admixtures. Genetic processes (genetic drift, founder effect, directional adaptation and hybridization) could have had a major role in the variability we observe in the European fossil record [8]. From the comparative study of the Atapuerca-SH large hominin sample, Martinón-Torres et al. [31] and Arsuaga et al. [13] also consider that more than one evolutionary lineage may have coexisted during the European Middle Pleistocene.

## Conclusions

We have presented a comparative morphological study of the Middle Pleistocene Montmaurin-LN mandible. Although geological studies and faunal remains point to a chronology coincident with MIS 7, it is premature to assume the chronology of the site in the absence of geochronological data. The lack of Large Cutting Tools and the Levallois technique in the still small sample of stone tools obtained from La Niche site opens the chronological range between MIS 9 to 6, the period corresponding to the Lower-Middle Palaeolithic transition [120].

This study indicates that the Montmaurin-LN specimen is related to the Neandertals of the Late Pleistocene. The external morphology of its molars is within the variability observed for the Atapuerca-SH mandibles and those of Neandertals. However, the bone element of the mandible shares only a few derived features with these hominins. Instead, the Montmaurin-LN specimen clusters with other European Middle Pleistocene specimens, as well with African and Asian mandibles of the same period. Although a geochronological study of La Niche cave is pending, it will be an interesting exercise to compare and contrast the predominant primitive morphology of this mandible with the quantitative results of future geochronological analyses. Thus, it will be possible to test these results against the different models, such as the accretion and the two-phase, to explain the variability of European Middle Pleistocene hominins. Considering the present evidence, we can hypothesize that the settlement of Europe was the result of several population waves, at different times, perhaps from the same common ancestor, as well as a complex history of wipe-outs and re-colonisations. We also hypothesize that genetic drift, founder effect, directional adaptations and hybridization did likely contribute to shape the evolution of these populations.

## Supporting information

S1 Table List of features used in the phenetic analysis. See S2 Table for the individual scoring.
(DOCX)

S2 Table Scoring of the features defined in S1 Table for different Pleistocene *Homo* mandibles. See Table 1 in the main text.
(DOCX)

S1 Fig Correspondence analysis biplots between dimension 1 and dimension 2. A biplot including all of the variables is represented on the left-hand side, while the one with a reduced number of variables is on the right. Light blue dots represent modern *H*. *sapiens*.
(TIF)

S2 Fig Percentage of inertia explained by the dimensions of the correspondence analysis using all variables in this study (left) and a reduced dataset (right). The horizontal red lines represent the threshold of an optimal dimensionality of the solution according to the average rule.
(TIF)

S3 Fig Dendrogram showing the results of the clustering analysis, when we include a reduced number of variables in order to include more specimens. Ward method and binary distance are used. Only bootstrap probabilities (in blue) over or equal to 70% are shown.
(TIFF)

## References

[1] Gabunia ML, Vekua A (1995) A Plio-Pleistocene hominid from Dmanisi, East Georgia, Caucasus. *Nature* 373: 509–512. doi: 10.1038/373509a0 7845461 10.1038/373509a0

[2] Carbonell E, Bermúdez de Castro JM, Arsuaga JL, Díez JC, Rosas A, Cuenca-Bescós G et al. (1995) Lower Pleistocene hominids and artifacts from Atapuerca-TD6 (Spain). Science 269: 826–830. doi: 10.1126/science.7638598 7638598 10.1126/science.7638598

[3] Ascenzi A, Biddittu I, Cassoli PF, Segre AG, Segre-Naldini E (1996) calvarium of late *Homo erectus* from Ceprano, Italy. J Hum Evol 31: 409–423. doi: 10.1006/jhev.1996.0069

[4] Bermúdez de Castro JM, Arsuaga JL, Carbonell E, Rosas A, Martínez I, Mosquera M (1997) A hominid from the Lower Pleistocene of Atapuerca, Spain: possible ancestor to Neandertals and modern humans. Science 276: 1392–1395. doi: 10.1126/science.276.5317.1392 9162001 10.1126/science.276.5317.1392

[5] Gabounia L, de Lumley MA, Vekua A, Lordkipanidze, de Lumley H (2002) Découverte d´un nouvel hominidé à Dmanissi (Trancaucasia, Géogie). C. R. Paleovol 1: 243–253. https://doi.org/10.1016/S1631-0683(02)00032-5.

[6] Carbonell E, Bermúdez de Castro JM, Parés JM, Pérez-González A, Ollé A, Mosquera M et al (2008). The first hominin of Europe. Nature 452: 465–469. doi: 10.1038/nature06815 18368116 10.1038/nature06815

[7] Manzi G, Magri D, Milli S, Palombo MR, Margari V, Celiberti V et al (2010) The new chronology of the Ceprano calvarium (Italy). J Hum Evol 59: 580–585. doi: 10.1016/j.jhevol.2010.06.010 20832845 10.1016/j.jhevol.2010.06.010

[8] Roksandic M, Mihailovic D, Mercier N, Dimitrijevic V, Morley MW, Rakocevic Z et al (2011). A human mandible (BH-1) from the Pleistocene deposits of Mala Balanica cave (Sicevo Gorge, Nis, Serbia). J Hum Evol 61: 186–196. doi: 10.1016/j.jhevol.2011.03.003 21507461 10.1016/j.jhevol.2011.03.003

[9] Cook J, Stringer CB, Currant HP, Schwarcz HP, Wintle AG (1982) A review of the chronology of the Europan Middle Pleistocene hominid record. Yearbook Phys Anthropol 25: 19–65.

[10] Dennell R, Martinón-Torres M, Bermúdez de Castro JM (2011) Hominid variability, climatic instability and population demography in Middle Pleistocene Europe. Quat Sci Rev 30: 1511–1524. doi: 10.1016/j.quascirev.2009.11.027

[11] Hublin J-J (1998) Climatic changes, paleogeography, and the evolution of the Neandertals In: Akazawa T, Aoki K, Bar-Yosef O, editors. New York: Plenum Press pp. 295–310.

[12] Hublin J-J (2009) The origin of Neandertals. Proc Natl Acad Sci, USA 106: 16022–16027. doi: 10.1073/pnas.0904119106 19805257 10.1073/pnas.0904119106 PMC2752594

[13] Arsuaga JL, Martínez I, Arnold LJ, Aranburu A, Gracia-Téllez A, Sharp WD et al (2014) Neandertal roots: Cranial and chronological evidence from Sima de los Huesos. Science 344: 1358–1363. doi: 10.1126/science.1253958 24948730 10.1126/science.1253958

[14] Stringer CB, Hublin J-J (1999) New age estimates for the Swanscombe hominid, and their significance for human evolution. J Hum Evol 37: 873–877. doi: 10.1006/jhev.1999.0367 10600325 10.1006/jhev.1999.0367

[15] Compton T, Stringer C (2015) The morphological affinities of the Middle Pleistocene hominin teeth from Pontnewydd Cave, Wales. J Quat Sci 30: 713–730. doi: 10.1002/jqs.2811

[16] De Lumley MA (2015) L´Homme de Tautavel. Un *Homo erectus* évolué. *Homo erectus tautavelensis*. L´Anthropologie 119: 303–348. doi: 10.1016/j.anthropo.2015.06.001

[17] Manzi G. (2004). Human evolution at the Matuyama-Bruhnes boundary. Evol Anthropol 13: 11–24. doi: 10.1002/evan.10127

[18] Daura J, Sanz M, Arsuaga JL, Hoffman DL, Quam RM, Ortega MC et al (2017) New Middle Pleistocene hominin cranium from Gruta da Aroeira (Portugal). Proc Natl Acad Sci, USA 314: 3397–3402. 3www.pnas.org/cgi/doi/10.1073/pnas.1619040114 10.1073/pnas.1619040114 PMC5380066 28289213

[19] Bermúdez de Castro JM, Martinón-Torres M, Rosell J, Blasco R, Arsuaga JL, Carbonell E (2016). Continuity *versus* discontinuity of the human settlement of Europe between the late Early Pleistocene and the early Middle Pleistocene. The mandibular evidence. Quat Sci Rev 153: 51–62. http://dx.doi.org/10.1016/j.quascirev.2016.10.010.

[20] Vallois HV (1955) La mandibule humaine pré-moustérienne de Montmaurin, Comptes rendus hebdomadaires des séances de l’Académie des sciences, Paris, 240: 1577–1579. 14379521

[21] Vallois HV (1956) The premousterian human mandible from Montmaurin. Am J Phys Anthropol 14: 319–324. 13362495 10.1002/ajpa.1330140224

[22] Crégut-Bonnoure E, Boulbes N, Guérin C, Penaud J, Tavoso A, Cammas R. (2010) Le contexte géomorphologique et faunique d l´homme de Montmaurin (Haute-Garonne). Préhistoires Méditerranéennnes 1, 3–85.

[23] Lisiecki LE, Raymo ME (2005) A Pliocene-Pleistocene stack of 57 globally distributed benthic d18O records. Paleoceanography 20, doi: 10.1029/2004PA001071

[24] Billy G, Vallois HV. (1977a) La mandibule pré-rissienne de Montmaurin, L’Anthropologie 81: 273–312.

[25] Billy G, Vallois HV. (1977b) La mandibule pré-rissienne de Montmaurin (suite), L’Anthropologie 81: 411–458.

[26] Rosas A (2001) Occurrence of Neanderthal features in mandibles from the Atapuerca-SH site. Am J Phys Anthropol 114: 74–91. doi: 10.1002/1096-8644(200101)114:1<74::AID-AJPA1007>3.0.CO;2-U 11150054 10.1002/1096-8644(200101)114:1<74::AID-AJPA1007>3.0.CO;2-U

[27] Mounier A, Marchak F, Condemi S (2009) Is *Homo heidelbergensis* a distinct species? New insight. J Hum Evol 56: 219–246 doi: 10.1016/j.jhevol.2008.12.006 19249816 10.1016/j.jhevol.2008.12.006

[28] Arsuaga JL, Martínez I, Gracia A, Lorenzo C (1997) The Sima de los Huesos crania (Sierra de Atapuerca, Spain). A comparative study. J Hum Evol 33: 219–281. doi: 10.1006/jhev.1997.0133 9300343 10.1006/jhev.1997.0133

[29] Hublin J-J (2009) The origin of Neandertals. Proc Natl Acad Sci, USA 106: 16022–16027. doi: 10.1073/pnas.0904119106 19805257 10.1073/pnas.0904119106 PMC2752594

[30] McDonald K, Martinón-Torres M, Dennell R, Bermúdez de Castro JM (2012) Discontinuity in the record for hominin occupation in south-western Europe: Implications for occupation of the middle latitudes of Europe. Quat Int 271: 84–97. https://doi.org/10.1016/j.quaint.2011.10.009.

[31] Martinón-Torres M, Bermúdez de Castro JM, Gómez-Robles A, Prado-Simón L, Arsuaga JL (2012) Morphological description and comparison of the dental remains from Atapuerca-Sima de los Huesos site (Spain). J Hum Evol 62: 7–58. doi: 10.1016/j.jhevol.2011.08.007 22118969 10.1016/j.jhevol.2011.08.007

[32] Lumley MA de (2015) L´homme de Tautavel. Un *Homo erectus* européen évolué. Homo erectus tautavelensis. L´Anthropologie 119: 303–348. https://doi.org/10.1016/j.anthro.2015.06.001.

[33] Bermúdez de Castro JM, Martinón-Torres M, Rosell J, Blasco R, Arsuaga JL, Carbonell E. (2016). Continuity *versus* discontinuity of the human settlement of Europe between the late Early Pleistocene and the early Middle Pleistocene. The mandibular evidence. Quat Sci Rev 153: 51–62. https://doi.org/10.1016/j.quascirev.2016.10.010.

[34] Boule M (1902) La caverne à ossements de Montmaurin (Haute-Garonne). L’Anthropologie 13: 305–319.

[35] Saint-Périer R (1922) Nouvelles recherches dans la caverne de Montmaurin (Haute-Garonne), L’Anthropologie 32: 193–202.

[36] Méroc L (1947) Montmaurin (La grotte de Coupe-Gorge). Gallia V: 193–194.

[37] Méroc L (1948) Les grottes de Montmaurin (Coupe- Gorge, Montmaurin, Zubiate, Les Abeilles), Gallia, Paris, 6: 409–412.

[38] Tavoso A (1982) Le cadre géochronologique de la mandibule de Montmaurin: examen des données disponibles, in: Premier congrès international de paléontologie humaine: section III, Nice, CNDP / CRDP: 96–97.

[39] Méroc L (1963) Les éléments de datation de la mandibule humaine de Montmaurin (Haute-Garonne) Bull Soc Géol, France 7: 508–515.

[40] Cammas R, Tavoso A. 1986 Nouveaux restes humains issus du remplissage de la Niche (Montmaurin, Haute-Garonne), C R Rendus Acad Sci, Paris (2) Paris, 302, 609–614.

[41] Gaillard C (1982) L´industrie lithique du Paléolithique inférieur et moyen de la grotte de Coupe-Gorge à Montmaurin (Haute-Garonne). Gallia Préhistoire 25: 79–105.

[42] Serra-Joulin D (2002) Les industries lithiques de la grotte de la Terrasse à Montmaurin (Haute.Garonne). Préhistoires Méditerranéennes 10–11: 5–26.

[43] Renault-Miskovsky J, Girard M. 1988 Palynologie des grottes de Montmaurin (Haute-Garonne) et du versant nord pyrénéen. Corrélations interséquentielles du Pléistocène moyen à l’Holocène [Palynology of Montmaurin caves (Haute-Garonne) and north-pyrenean slope, Intersequential correlations from middle Pleistocene to Holocene.]. Quaternaire 9: 185–201.

[44] Aguirre E, Basabe JM, Torres T (1976) Los fósiles humanos de Atapuerca (Burgos): nota preliminar. ZEPHYRUS 26–27: 489–511. http://revistas.usal.es/index.php/0514-7336/article/view/499.

[45] Aguirre E, de Lumley MA (1977) Fossil men from Atapuerca, Spain: Their bearing on human evolution in the Middle Pleistocene. J Hum Evol 6: 681–688. https://doi.org/10.1016/S0047-2484(77)80094-8.

[46] Rosas A, Bermúdez de Castro JM (1998) The Mauer mandible and the evolutionary significance of *Homo heidelbergensis*. Geobios 31: 687–697. https://doi.org/10.1016/S0016-6995(98)80055-7.

[47] Tobias PV (1991) Olduvai Gorge. Volume 4: the skulls, endocasts and teeth of *Homo habilis*. Cambridge: Cambridge University Press.

[48] Schrenk F, Bromage TG, Betzler CG, Ring U, Juwayeyi Y (1993) Oldest *Homo* and Pliocene biogeography of the Malawi Rift. Nature 365: 833–836. doi: 10.1038/365833a0 8413666 10.1038/365833a0

[49] Wood BA (1991) Koobi Fora research Project Volume 4 Hominid cranial remains from Koobi Fora. Oxford: Clarendon Press.

[50] Day MH, Leakey REF (1973) New evidence of the genus *Homo* from East Rudolf, Kenya. I. Am J Phys Anthropol 39: 341–354. doi: 10.1002/ajpa.1330390303 4753133 10.1002/ajpa.1330390303

[51] Leakey REF, Wood BA (1973) New evidence of the genus *Homo* from East Rudolf, Kenya (II). Am J Phys Anthropol 39: 355–368. doi: 10.1002/ajpa.1330390304 4753134 10.1002/ajpa.1330390304

[52] Leakey REF, Leakey MG, Beherensmeyer AK (1978) The hominid catalogue In: Leakey MG, Leakey REF, editors. Koobi Fora research Project, volumen 1: the fossil hominids and an introduction to their context 1968–1974. Oxford: Clarendon Press pp. 86–182.

[53] Walker A, Leakey REF (1993) The skull In: Walker A, Leakey R, (Eds.). The Nariokotome *Homo erectus* skeleton. Berlin: Springer-Verlag pp. 63–94.

[54] Suwa G, Asfaw B, Haile-Selassie Y, White T, Katoh S, WoldeGabriel G et al (2007). Early Pleistocene *Homo erectus* fossils from Konso, southern Ethiopia. Athropol Sci 115: 133–151. http://doi.org/10-1537/ase.061203.

[55] Leakey LBS, Tobias PV, Martyn JE, Leakey REF (1969) An Acheulan industry with prepared core technique and the discovery of a contemporary hominid mandible at Lake Baring, Kenya. Proc Prehist Soc 25: 48–76.

[56] Wood BA, Van Noten FL (1986) Preliminary observations on the BK 8518 mandible from Baring, Kenya. Am J Phys Anthropol 69: 117–127. doi: 10.1002/ajpa.1330690113 3080897 10.1002/ajpa.1330690113

[57] Sausse F (1975) Mandibule de la carrière Thoma 1 (Casablanca). Anthropologie 79: 81–112.

[58] Rightmire GP (1990) The Evolution of *Homo erectus*. Comparative Anatomical Studies of an Extinct Human Species. Cambridge: Cambridge University Press.

[59] Kaifu Y, Aziz F, Baba H (2005) Hominid mandibular remains from Sangiran: 1952–1986 Collection. Am J Phys Anthropol 128: 497–519. doi: 10.1002/ajpa.10427 15761881 10.1002/ajpa.10427

[60] Kaifu Y, Baba H, Aziz F, Indriati E, Schrenk F, Jacob T (2005) Taxonomic affinities and evolutionary history of the Early Pleistocene Hominid of Java. Dentognathic evidence. Am J Phys Anthropol 128: 709–726. doi: 10.1002/ajpa.10425 15761880 10.1002/ajpa.10425

[61] Weidenreich F (1936) The mandibles of *Sinanthropus pekinensis*: a comparative study. Acta Palaeontologica Sinica (New Series) 7:1–162.

[62] Chang C-H, Kaifu Y, Takai M, Kono MT, Grün R., Matsu´ura S et al (2015) The first archaic *Homo* from Taiwan. Nature Com 6:6037 doi: 10.1038/ncomms7037 25625212 10.1038/ncomms7037 PMC4316746

[63] Wu L, Martinón-Torres M, Kaifu Y, Xiujie Wu, Kono R-T, Chang C-H et al (2017) A mandible from the Middle Pleistocene Hexian site and its significance in relation to the variability of Asian *Homo erectus* Am J Phys Anthropol. doi: 10.1002/ajpa.23162 28109118 10.1002/ajpa.23162

[64] Rosas A (1987) Two new mandibular frgaments from Atapuerca/Ibeas (SH site). A reassessment of the affinities of the Ibeas mandibles simple. J Hum Evol 16: 417–427. https://doi.org/10.1016/0047-2484(87)90070-4.

[65] Rosas A (1995) Seventeen new mandibular specimens from the Atapuerca (Ibeas Middle Pleistocene Hominids sample. J Hum Evol 28: 533–559. https://doi.org/10.1006/jhev.1995.1041.

[66] Heim JL (1976) Les hommes de la Ferrassie. Tome 1. Le gisement, les squelettes adultes (crânes et squelettes du tronc) In: Archives de l´Institut de Paléontologie Humaine. Mémoire, vol. 35 Paris: Masson.

[67] Trinkaus E (1983) The Shanidar Neanderthals. New York: Academic Press.

[68] Alcázar de Velasco A, Arsuaga JL, Martínez I, Bonmatí A (2011) The Bañolas human mandible revisited (Gerona, Spain). Bol R Soc Esp Hist Nat, Geol 105: 99–10.

[69] Hershokvitz L, Speirs MS, Frayer D, Nadel D, Wish-Betraz S, Arensburg B (1995) Ohalo II H2: a 19,000-year-old skeleton from a water-logged site at the sea of Galilee, Israel. Am J Phys Anthropol 96: 215–234. doi: 10.1002/ajpa.1330960302 7785722 10.1002/ajpa.1330960302

[70] Trinkaus E, Moldovan O, Milota S, Bilgar A, Sarcina S, Athreya S (2003) An early modern human from the Petera cu Oase, Romania. Proc Natl Acad Sci, USA 20: 11231–11236. doi: 10.1073/pnas.2035108100 10.1073/pnas.2035108100 PMC208740 14504393

[71] Sneath PHA (1995) Thirty years of numerical taxonomy. Syst Biol 44: 281–298. https://doi.org/10.1093/sysbio/44.3.281.

[72] Stringer CB, Hublin JJ, Vandermeersch B. 1984 The origin of anatomically modern humans in Western Europe In: Smith FH, Spencer F, editors. The origins of modern humans: a world survey of the fossil evidence. New York: Liss p 51–135.

[73] Condemi S. 1991 Circeo I and variability among classic Neanderthals In: Piperno M, Scichilone G, editors. The Circeo 1 Neanderthal skull: studies and documentation. Rome: Instituto Poligrafico e Zecca dello Stato p 339–353.

[74] Trinkaus E. (1993) Variability in the position of the mandibular mental foramen and the identification of Neandertal apomorphies. Rivista di Antropologia (Roma) 71:259–274.

[75] Rak Y, Ginzberg A, Geffen E. (2002) Does *Homo neanderthalensis* play a role in modern human ancestry? The mandibular evidence. Am J Phys Anthropol 119:199–204. doi: 10.1002/ajpa.10131 12365031 10.1002/ajpa.10131

[76] Wolpoff MH, Frayer DW (2005) Unique ramus anatomy for Neandertals? Am J Phys Anthropol 128: 245–251. doi: 10.1002/ajpa.10432 15816039 10.1002/ajpa.10432

[77] Nicholson E, Harvati K (2006). Quantitative analysis of human mandibular shape using three-dimensional geometric morphometrics. Am J Phys Anthropol 131: 368–383. doi: 10.1002/ajpa.20425 16617436 10.1002/ajpa.20425

[78] Lefêvre J (1973) Etude odontologique des hommes de Muge. Bull Mém Soc Anthrop, Paris 12: 301–333.

[79] Brothwell DR (editor) (1963). Dental Anthropology. New York, Pergamon Press.

[80] Turner II CG, Nichol CR, Scott GR (1991) Scoring procedures for key morphological traits of the permanent dentition: the Arizona State University dental anthropology system In: Kelley M, Larsen C, editors. Advances in dental anthropology. Wiley-Liss, New York, pp. 13–31.

[81] Scott GR, Turner CG II (1997) The Anthropology of Modern Human Teeth: Dental Morphology and Its Variation in Recent Human Populations. Cambridge University Press, Cambridge.

[82] Molnar S (1971) Human tooth wear, tooth function and cultural variability. Am J Phys Anthropol 34: 175–189. doi: 10.1002/ajpa.1330340204 5572602 10.1002/ajpa.1330340204

[83] White TD, Johanson DC, Kimbel WH (1981) *Australopithecus africanus*: its phyletic position reconsidered. S Afr J Sci 77: 445–470.

[84] Howell FC (1960) European and Northwest African Middle Pleistocene hominids. Curr Anthropol 1: 195–232.

[85] Wu L, Jin C-Z, Zhang Y-Q, Cai Y-C, Xing S, Wu X-J et al (2010). Human remains from Zhirendong, South China, and modern human emergence in East Asia. Proc Natl Acad Sci, USA 107: 19201–19206. doi: 10.1073/pnas.1014386107 20974952 10.1073/pnas.1014386107 PMC2984215

[86] Schwartz JH, Tattersall I (2000) The human chin: what is it and who has it? J Hum Evol 38: 367–409. doi: 10.1006/jhev.1999.0339 10683306 10.1006/jhev.1999.0339

[87] Rak Y (1986) The Neanderthal: a new look at an old face. J Hum Evol 15: 151–164. https://doi.org/10.1016/S0047-2484(86)80042-2.

[88] Condemi S (1991) Some considerations concerning Neandertal features and the presence of Neandertals in the Near East. Rivista di Antropologia (Roma) LXIX: 27–38.

[89] Rosas A, Bermúdez de Castro JM, Aguirre E (1991) Mandibules et dents d´Ibeas (Espagne) dans le contexte de l´evolution humaine en Europa. L´Anthropologie 4: 89–112.

[90] Franciscus RG, Trinkaus E (1995) Determinants of retromolar space presence in Pleistocene *Homo* mandibles. J Hum Evol 28: 577–595. https://doi.org/10.1006/jhev.1995.1043.

[91] Trinkaus E (1987) The Neandertal face: evolutionary and functional perspectives on a recent hominid face. J Hum Evol 16: 429–443. https://doi.org/10.1016/0047-2484(87)90071-6.

[92] Rosas A, Bermúdez de Castro JM (1999) The ATD6-5 mandibular specimen from Gran Dolina (Atapuerca, Spain). Mophological study and phylogenetic implications. J Hum Evol 37: 567–590. doi: 10.1006/jhev.1999.0340 10497000 10.1006/jhev.1999.0340

[93] Trinkaus E (2006) Modern human versus Neandertal evolutionary distinctiveness. Curr Anthropol 47:597–620.

[94] Weaber T (2009) The meaning of Neandertal skeletal morphology. Proc Natl Acad Sci, USA 106: 16028–16033. doi: 10.1073/pnas.0903864106 19805258 10.1073/pnas.0903864106 PMC2752516

[95] Rak Y, Kimbel WH, Hovers E (1994) A neandertal infant from Amud Cave. J Hum Evol 26: 313–324. https://doi.org/10.1006/jhev.1994.1019.

[96] Bermúdez de Castro JM, Quam T, Martinón-Torres M, Martínez I, Gracia-Téllez A, Arsuaga JL, et al (2014). The medial pterygoid tubercle in the Atapuerca Early and Middle Pleistocene mandibles. Evolutionary implications. Am J Phys Anthropol 156: 102–109. doi: 10.1002/ajpa.22631 25279839 10.1002/ajpa.22631

[97] Wu X-J, Trinkaus E (2013) The Xujiayao 14 mandibular ramus and Pleistocene *Homo* mandibular variation. C R Palevol 13: 333–341. https://doi.org/10.1016/j.crpv.2013.10.002.

[98] Rosas A, Bermudez de Castro JM (1998) On the taxonomic affinities of the Dmanisi mandible (Georgia). Am J Phys Anthropol 107: 145–162. doi: 10.1002/(SICI)1096-8644(199810)107:2<145::AID-AJPA2>3.0.CO;2-U 9786330 10.1002/(SICI)1096-8644(199810)107:2<145::AID-AJPA2>3.0.CO;2-U

[99] Carbonell E, Bermúdez de Castro JM, Arsuaga JL, Allue E, Bastir M, Benito A et al (2005) An early Pleistocene hominin mandible from Atapuerca-TD6, Spain. Proc Natl Acad Sci, USA 102: 5674–5678. doi: 10.1073/pnas.0501841102 15824320 10.1073/pnas.0501841102 PMC556125

[100] Wolpoff MH (1979) The Krapina dental remains. Am J Phys Anthropol 50: 67–114. doi: 10.1002/ajpa.1330500110 736116 10.1002/ajpa.1330500110

[101] Bailey SE (2002) A closer look at Neanderthal postcanine dental morphology: the mandibular dentition. Anat Rec 269: 148–156. doi: 10.1002/ar.10116 12124901 10.1002/ar.10116

[102] Bailey SE, Skinner MM, Hublin J-J (2011) What lies beneath? An evaluation of the mid-trigonid crest dental trait based on both dentine and enamel expression. Am J Phys Anthropol 145: 505–518. doi: 10.1002/ajpa.21468 21312178 10.1002/ajpa.21468

[103] Irish JD (1993) Biological affinities of late Pleistocene through modern African Aboriginal populations: the dental evidence. Ph.D. Dissertation, Arizona State University.

[104] Rosas A (1997). A gradient of size and shape for the Atapuerca sample and Middle Pleistocene hominid variability. J Hum Evol 33: 319–331. doi: 10.1006/jhev.1997.0138 9300345 10.1006/jhev.1997.0138

[105] Wagner GA, Krbetschek M, Degering D, Bahain J-J, Shao Q, Falguères et al (2010). Radiometric dating of the type-site for *Homo heidelbergensis* at Mauer, Germany. Proc Natl Acad Sci, USA, 107: 19706–19730. doi: 10.1073/pnas.1012722107 21041630 10.1073/pnas.1012722107 PMC2993404

[106] Rosas A, Bastir M, Martínez-Maza C, García-Tabernero A, Lalueza-Fox C (2006) Inquiries into Neanderthal craniofacial development and evolution: “accretion” versus “organismic” models In: Harvati K, Harrison T (Eds.). Neanderthals Revisited: New Approaches and Perspectives, New York: Springer pp. 37–70.

[107] Dean D, Hublin J-J, Holloway R, Ziegler R (1998) On the phylogenetic position of the pre-Neandertal specimen from Reilingen, Germany. J Hum Evol 40: 485–508. https://doi.org/10.1006/jhev.1998.0214. 10.1006/jhev.1998.0214 9614635

[108] Manzi, Mallegni F, Ascenzi A (2001). A cranium for the earliest Europeans: Phylogenetic position of the hominid from Ceprano, Italy. Proc Natl Acad Sci, USA 98: 10011–10016. doi: 10.1073/pnas.151259998 11504953 10.1073/pnas.151259998 PMC55569

[109] Bermúdez de Castro JM, Martinón-Torres M, Sarmiento S, Lozano M (2003) Gran Dolina-TD6 *versus* Sima de los Huesos dental samples from Atapuerca: Evidence of discontinuity in the European Pleistocene population? J Archaeol Sci 30: 1421–1428. http://doi.org/10.1016/S0305-4403(03)00036-0

[110] Dennell R., Martinón-Torres M., Bermúdez de Castro J.M. 2011 Hominid variability, climatic instability and population demography in Middle Pleistocene Europe. Quat Sci Rev 30: 1511–1524. https://doi.org/10.1016/j.quascirev.2009.11.027

[111] Hughes JK, Haywood A, Mithen SJ, Sellwodd BW, Valdes PJ (2007) Investigating early hominin dispersals patterns: developing a framework for climate data investigation. J Hum Evol 53: 465–474. doi: 10.1016/j.jhevol.2006.12.011 17923151 10.1016/j.jhevol.2006.12.011

[112] Carrión JS, Rose J, Stringer C (2011) Early human evolution in the Western Palearctic: ecological scenarios. Quat Sci Rev 30: 1281–1295. http://doi.org/10.1016/j.quascirev.2011.04.003

[113] Bermúdez de Castro JM, Martinón-Torres M (2013) A new model for the evolution of the human Pleistocene populations of Europe. Quat Int 295: 102–112. http://doi.org/10.1016/j.quaint.2012.02.036

[114] Finlayson C, Carrión JS (2007) Rapid ecological turnover and its impact on Neanderthal and other human populations. Trends Ecol Evol 22: 213–222. doi: 10.1016/j.tree.2007.02.001 17300854 10.1016/j.tree.2007.02.001

[115] Blain HA, Bailon S, Cuenca-Bescós G, Arsuaga JL, Bermúdez de Castro JM (2009) Long-term climate record inferred from early-middle Pleistocene amphibian and squamate reptile assemblages at the Gran Dolina Cave, Atapuerca, Spain. J Hum Evol 56: 55–65. doi: 10.1016/j.jhevol.2008.08.020 18986681 10.1016/j.jhevol.2008.08.020

[116] Shea JJ (2008) Transitions or turnovers? Climatically-forced extinctions of *Homo sapiens* and Neanderthals in the East Mediterranean Levant. Quat Sci Rev 27: 2253–2270. http://doi.org/10.1016/j.quascirev.2008.08.015

[117] Agustí J, Blain H-A, Cuenca-Bescós G, Bailon S (2009) Climate forcing of first hominid dispersal in Western Europe. J Hum Evol 57: 815–821. doi: 10.1016/j.jhevol.2009.06.005 19683790 10.1016/j.jhevol.2009.06.005

[118] Cuenca-Bescós G, Melero-Rubio M, Rofes J, Martínez I, Arsuaga JL, H-A et al (2011) The EarlyeMiddle Pleistocene environmental and climatic change and the human expansion in Western Europa: a case study with small vertebrates (Gran Dolina, Atapuerca, Spain). J Hum Evol 60: 481–491. doi: 10.1016/j.jhevol.2010.04.002 20573376 10.1016/j.jhevol.2010.04.002

[119] Leroy SAG, Arpe K, Mikolajewicz U (2011) Vegetation context and climatic limits of the Early Pleistocene hominin dispersal in Europe. Quat Sci Rev 30: 1448–1463. http://doi.org/10.1016/j.quascirev.2010.01.017

[120] Hérisson D, Brenet M, Cliquet D, Moncel MH, Richter J, Scott B et al (2016). The emergence of the Middle Palaeolithic in north-western Europe and its southern fringes. Quat Int 411: 233–283. https://doi.org/10.1016/j.quaint.2016.02.049

