# Supplementary material for: A reassessment of the Montmaurin-La Niche mandible (Haute Garonne, France) in the context of European Pleistocene human evolution
Source: PLoS One. 2018 Jan 16;13(1):e0189714. doi: 10.1371/journal.pone.0189714 (PMC5770020; doi:10.1371/journal.pone.0189714)
Supplement: S1 Table — See S2 Table for the individual scoring. (DOCX) [file pone.0189714.s001.docx]

S1 Table. List of features used in the phenetic analysis. See S2 Table for the individual scoring.

A. Structures related to the bony chin: tuber symphyseos, lateral tubercles, central keel, mental fossae, and thickening of the inferior margin.

1: Absent or weakly develop of some of these structures.

2: Present and strongly developed.

B- Fossae digastrica. Direction in which the fossae are facing

1: Downward

2: Downward-backward

3: Backward

C- Alveolar margin orientation toward inferior margin.

1: Steep

2: Parallel or slowly inclined

D- Place of the foramen mentale toward the tooth row

1: P3-P4, P4

2: P4-M1, M1

E- Place of the lateral prominence along the tooth row

1: M1, M1-M2, M2

2: M2-M3

3: M3

F- Relationship between the anterior ramus rim and M3 in norma lateralis.

1: Covered

2: Partially covered

3: Uncovered

G- Inclination of the retromolar area

1: Vertical

2: Inclined

3: Horizontal

H- Fossa masseterica: Depth of a variably expressed hollowing on the lateral surface of the gonian angle.

1: Deep

2: Shallow or flat

I- Gonion profile

1: Expanded

2: Regular

3: Truncated

J- Position of the mandibular notch´deepest point.

1: Medial

2: Posterior

K- Condyle height relative to the coronoid.

1: Lower

2: Equal

3: Higher

L- Position of the junction between mandibular notch and the condyle articular surface.

1: Lateral

2: Medial

M- Pterygoid fossa

1: Shallow

2: Deep

N- Orientation of the mylohyoid line

1: Parallel, subparallel, inclined

2: Diagonal

O- Planum alveolare.

1: Well developed and inclined

2: Vertical or almost vertical
