# Supplementary material for: A reassessment of the Montmaurin-La Niche mandible (Haute Garonne, France) in the context of European Pleistocene human evolution
Source: PLoS One. 2018 Jan 16;13(1):e0189714. doi: 10.1371/journal.pone.0189714 (PMC5770020; doi:10.1371/journal.pone.0189714)
Supplement: S2 Table — See Table 1 in the main text. (DOCX) [file pone.0189714.s002.docx]

S2 Table. Scoring of the features defined in S1 Table in different Pleistocene *Homo* mandibles. See Materials in the main text.

Specimen A B C D E F G H

D2735 1 1 1 1 1 2 1 1

D2600 1 1 1 1 2 2 2 2

Sangiran 1b 1 1 2 1 1 2 2 -

Sangiran 9 1 1 2 1 1 - - 1

Sangiran 22 1 1 - 1 1 - - 2

Penghu 1 1 2 2 1 1 1 2 1

KNM-ER 992 1 1 2 1 1 2 2 1

ATD6-96 1 1 2 1 1 2 2 2

Tighenif 1 1 2 2 1 1 2 3 1

Tighenif 2 1 3 2 1 2 2 2 2

Tighenif 3 1 1 2 1 2 3 2 2

Mauer 1 1 1 2 3 2 3 2

Arago II 1 1 2 2 3 3 3 1

Arago XIII 1 1 2 2 2 3 2 1

**Montmaurin -LN** 1 2 2 1 3 2 3 2

AT-300 1 1 1 2 3 3 2 1

AT-505^1^ 1 1 2 2 3 3 3 1

AT-605 1 2 2 2 3 3 3 1

AT-607 (immat.) 1 2 2 2 3 3 3 2

AT-888 1 2 2 2 3 3 3 1

AT-950 1 1 2 2 3 3 3 1

Ehringsdorf F 1 1 2 2 3 3 3 1

Zhoukudian H1 1 2 2 2 2 2 2 1

Zhukoudian G1 1 - 2 1 2 2 2 1

Krapina J 1 1 2 2 3 3 3 1

Krapina H 1 2 2 2 3 - - -

Krapina G 1 1 2 2 3 3 3 2

Spy 1 1 3 1 2 3 3 3 2

Shanidar 1 1 2 1 2 3 3 3 2

Regourdou 1 1 1 2 2 3 3 3 2

Bañolas 1 3 2 2 3 3 2 2

La Ferrasie 1 1 1 2 2 3 3 3 2

La Quina H5 1 2 2 2 3 3 3 1

Amud 1 1 2 2 2 3 3 3 2

Zafarraya 2 1 2 2 2 2 3 3 2

Qafzeh 9 2 2 1 1 2 2 2 2

Skhül 5 2 2 1 1 2 3 3 2

Cro-Magnon 1 2 2 1 1 1 2 3 2

Abri Pataud 1 2 1 2 1 1 1 2 2

Ohalo 2 H2 2 3 2 1 1 2 2 2

Pestera cu Oase 2 - 2 1 2 2 2 2

1969-26-2 2 3 1 2 3 2 2 2

1969-27-4 2 3 1 1 1 3 2 2

1969-22-2 2 3 1 2 3 3 2 1

1969-27-2 2 3 2 1 2 2 2 2

1969-28-2 2 3 1 1 2 2 2 2

1969-39-2 2 3 1 1 1 2 2 2

1969-36-2 2 3 1 1 3 3 2 2

1969-30-4 2 3 1 1 1 2 2 2

1969-32-2 2 3 1 1 2 3 2 2

1969-41-16 2 3 1 2 2 2 2 2

1969-41-12 2 3 1 1 2 3 2 2

1969-41-2 2 3 1 1 1 2 2 2

1969-41-8 2 3 1 2 2 3 2 2

1969-41-14 2 3 2 1 2 2 2 2

1969-40-6a 2 2 2 1 1 2 2 1

1969-40-6b 2 2 2 1 2 2 2 1

1969-112-2 2 3 1 1 1 2 2 1

1972-23-2 2 3 1 2 2 2 2 2

1955-101-2 2 2 2 1 3 3 2 2

1955-98-2 2 2 2 1 1 2 3 1

1969-41-10 2 2 2 1 3 3 2 2

1969-15-2 2 3 2 1 1 2 2 2

1955-111-2 2 2 1 1 2 3 2 2

1969-15-11 2 2 1 1 1 2 2 2

1969-17-2 2 2 1 1 2 3 2 2

1955-111 2 2 1 1 2 2 2 2

1955-110-2 2 3 1 1 3 3 2 2

Specimen I J K L M N O

D2735 2 2 - 1 1 1 1

D2600 - - 1 1 1 2 1

Sangiran 1b - - - - - 1 1

Sangiran 9 - - - - 2 - 1

Sangiran 22 - - - - 1 1 1

Penghu 1 3 2 - 1 1 1 -

KNM-ER 992 1 1 - - 1 1 1

ATD6-96 2 1 2 1 1 1 2

Tighenif 1 - - - - 1 1 1

Tighenif 2 - 1 - 2 1 1 1

Tighenif 3 3 2 1 1 2 1 1

Mauer 3 2 2 1 1 1 1

Arago 2 2 - - 1 2 1 1

Arago 13 2 1 2 2 1 1 1

**Montmaurin** 2 1 2 2 2 1 1

AT-300 - 1 2 1 1 2 1

AT-505 2 2 - 2 1 2 1

AT-605 2 2 1 2 1 2 1

AT-607 2 1 2 2 1 2 1

AT-888 2 - 2 2 1 2 1

AT-950 2 2 2 2 1 1 1

Ehringsdorf F - - - - 1 1 1

Zhoukudian H1 1 1 1 1 1 1 1

Zhoukoudian G1 2 1 1 1 1 1 2

Krapina J 3 2 1 2 2 2 2

Krapina H - - - - 1 2 1

Krapina G - - - - 2 2 1

Spy 1 - - - 1 2 2 2

Shanidar 1 3 2 1 2 2 2 2

Regourdou 1 3 2 1 2 2 2 2

Bañolas 2 - - 1 1 1 2

La Ferrasie 1 3 2 - 2 2 2 2

La Quina H5 3 2 1 2 1 2 2

Amud 1 3 2 1 2 1 1 2

Zafarraya 2 3 2 1 2 2 2 2

Qafzeh 9 2 1 1 1 2 1 2

Skhül 5 2 1 2 1 1 1 2

Cro-Magnon 1 2 - 1 - 1 1 1

Abri Pataud 1 2 1 3 1 1 1 2

Ohalo 2 H2 1 2 1 1 1 1 2

Pestera cu Oase 1 1 2 1 1 1 2

1969-26-2 2 1 2 1 1 1 2

1969-27-4 2 1 2 1 2 1 2

1969-22-2 2 1 2 1 1 1 2

1969-27-2 2 1 2 1 1 1 2

1969-28-2 2 1 2 1 1 1 2

1969-39-2 2 1 2 1 1 2 2

1969-36-2 2 1 2 1 1 2 2

1969-30-4 2 1 2 1 1 1 2

1969-32-2 2 1 2 1 2 1 2

1969-41-16 2 1 2 1 1 1 2

1969-41-12 2 1 2 1 2 1 2

1969-41-2 3 1 2 1 1 1 2

1969-41-8 2 1 2 1 1 1 2

1969-41-14 2 1 2 1 1 1 2

1969-40-6a 2 1 2 1 2 1 2

1969-40-6b 2 1 2 1 1 2 2

1969-112-2 2 1 2 1 1 2 2

1972-23-2 2 1 2 1 1 1 2

1955-101-2 2 1 2 1 1 2 2

1955-98-2 2 1 2 1 1 1 2

1969-41-10 2 1 2 1 1 1 2

1969-15-2 2 1 2 1 1 1 2

1955-111-2 2 1 2 1 1 1 2

1969-15-11 2 1 2 1 1 1 2

1969-17-2 2 1 2 1 1 1 2

1955-111 2 1 2 1 1 1 2

1955-110-2 3 1 2 1 2 2 2

1: Some of the Sima de los Huesos specimens are formed by several fragments, which were recovered during different field seasons. In this Table we note only the first recovered fragment.
