# Supplementary figures and images for: A reassessment of the Montmaurin-La Niche mandible (Haute Garonne, France) in the context of European Pleistocene human evolution
Source: PLoS One. 2018 Jan 16;13(1):e0189714. doi: 10.1371/journal.pone.0189714 (PMC5770020; doi:10.1371/journal.pone.0189714)

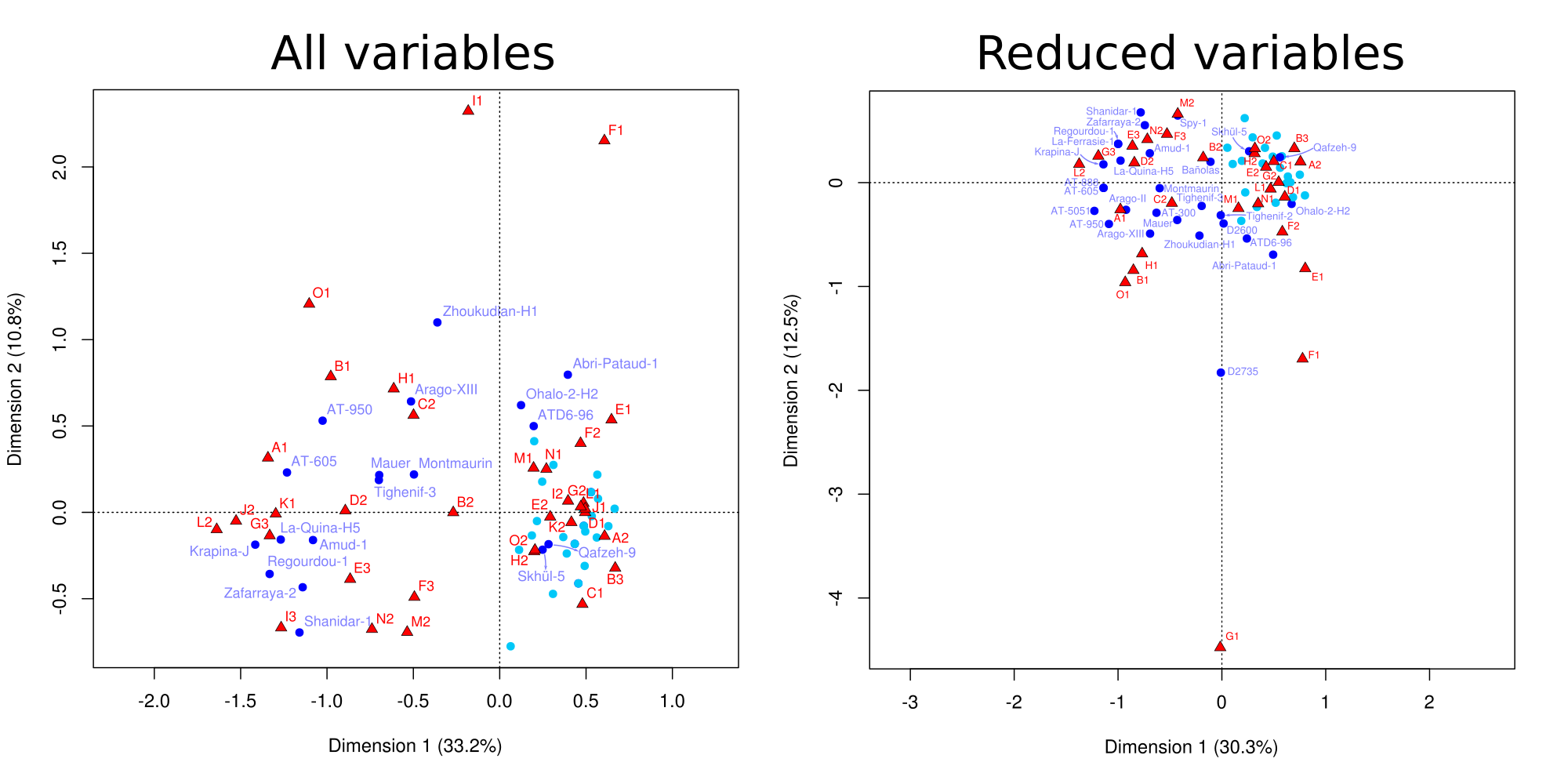

Supplement: S1 Fig — A biplot including all of the variables is represented on the left-hand side, while the one with a reduced number of variables is on the right. Light blue dots represent modern H. sapiens. (TIF) [file pone.0189714.s003.tif]

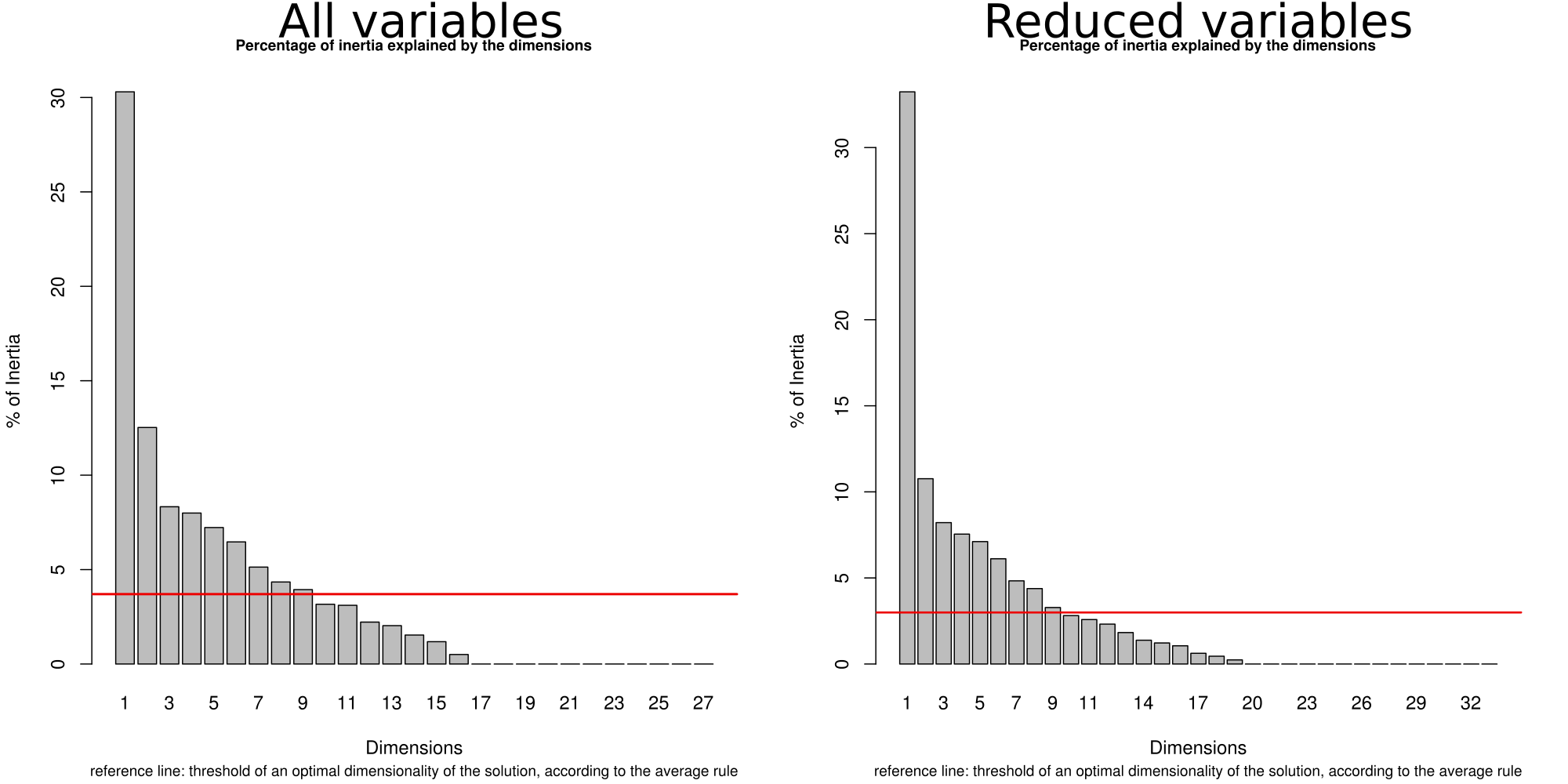

Supplement: S2 Fig — The horizontal red lines represent the threshold of an optimal dimensionality of the solution according to the average rule. (TIF) [file pone.0189714.s004.tif]

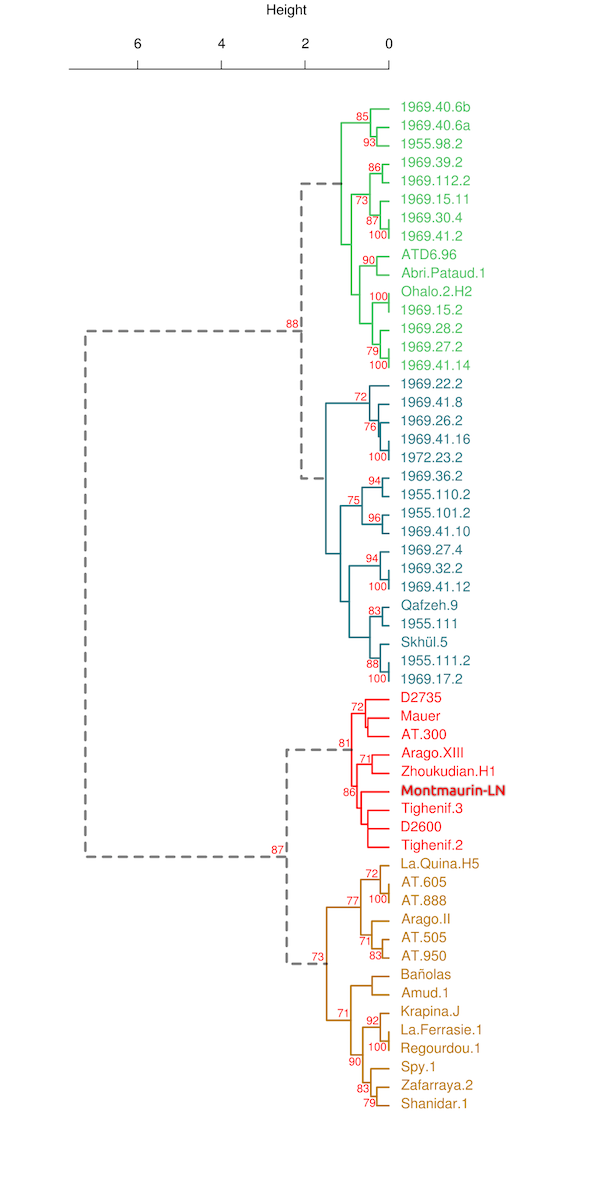

Supplement: S3 Fig — Ward method and binary distance are used. Only bootstrap probabilities (in blue) over or equal to 70% are shown. (TIFF) [file pone.0189714.s005.tiff]
